# Experimental analysis of elevated temperature and soiling loss on the rooftop PV modules performance under composite climatic conditions

**DOI:** 10.1038/s41598-025-25846-z

**Published:** 2025-11-26

**Authors:** Deepak Yadav, Satish Kumar Yadav, Vishnu Varma, Sidra Khatoon, Jyotsna Singh, Rajendra Bahadur Singh, S. M. Mozammil Hasnain, Prabhu Paramasivam, Mitiku Adare Tufa

**Affiliations:** 1 Department of Physics, University of Lucknow, Lucknow, U.P. India; 2 Institute of New and Renewable Energy, University of Lucknow, Lucknow, U.P. India; 3 Greenko Group, Hyderabad, 500081 Telangana India; 4 Marwadi University Research Centre, Department of Mechanical Engineering, Faculty of Engineering & Technology, Marwadi University, Rajkot, 360003 Gujarat India; 5 Centre for Research Impact & Outcome, Chitkara University Institute of Engineering and Technology, Chitkara University, Rajpura, 140401 Punjab India; 6 Department of Civil Engineering, Salale University, Salale, Fiche, 245 Ethiopia

**Keywords:** Solar PV system, Ambient temperature, Efficiency, Cell temperature, Soiling, Climate sciences, Energy science and technology, Engineering, Environmental sciences

## Abstract

On-field performance of a solar PV plant completely relies on solar radiation intensity and its availability. During operation, the output of a solar PV module varies from its rated capacity due to weather parameters such as ambient temperature, humidity, rainfall, wind, and dust. The primary objective of the study is to experimentally evaluate the combined effect of elevated temperature, soiling, and wind-driven convective cooling on rooftop PV performance under composite climatic conditions of North India, which are less explored compared to arid or temperate regions. During the investigation, the effects of elevated operating temperature, daily soiling accumulation, and wind-driven convective cooling on a 500 Wp rooftop PV setup monitored at five-minute intervals throughout June. The results reveal that elevated cell temperatures peaking at 64.0 °C caused an average daily efficiency reduction of 12.0%, with a 9.6% decline in output power directly attributable to temperature effects. Maximum soiling loss was calculated about 0.47% per day with a total monthly loss of 10.2% with some occasional showers cleaning the module by the end of the month. Convective heat loss from the roof mounted PV array was found to be 16.19% higher than a simulated ground-mounted system due to elevated wind exposure, contributing to a 2.27% improvement in energy yield. This study is based on a pilot-scale rooftop installation monitored for a single month. While results were normalized for broader applicability, the scope remains limited, and findings should be interpreted accordingly. The findings highlight that rooftop PV system is simultaneously affected by thermal and soiling effects, and both require explicit consideration at the design stage. Enhanced wind exposure can provide a natural and maintenance-free cooling benefit over ground-mounted systems. Site-specific cleaning schedules are essential to prevent daily dust accumulation.

## Introduction

The rapid pace of urbanization, industrialization, and population growth has significantly escalated the global demand for energy. This demand has traditionally been met through fossil fuels such as coal, oil, and natural gas, which are not only limited in availability but also major contributors to carbon emissions and global warming^1^. This has intensified concerns over environmental degradation and the urgent need to transition towards more sustainable energy sources. As a result, renewable energy technologies have gained global attention, among which solar energy stands out due to its abundance, environmental friendliness, and ease of harnessing^2^. Solar energy, in particular, offers a clean and sustainable solution to meet modern energy needs, aligning with the global goals of affordable and clean energy (SDG7) and climate action (SDG13)^3^. The advancements in solar technologies have made it one of the most promising alternatives to fossil fuels, supporting both energy security and environmental sustainability.

The weather parameters (temperature, wind speed, humidity, dust) along with geographical factors (latitude, longitude) affect the yield of a solar PV system^4^. Hence, performance analysis of a SPV module in the real environment provides crucial information required for forecasting the energy output of a photovoltaic power plant. The output of the SPV module gets reduced with the rise of ambient temperature^5^. *A study has reported that the generation efficiency of SPV plants decreases from about 12.05% to 6.60% as the temperature increases from 36 °C to 45 °C. Furthermore*,* with temperature rising beyond 45 °C*,* a constant decline in efficiency was observed*,* with monocrystalline solar photovoltaic cells displaying only 2.37% efficiency at 58 °C*,* and a maximum efficiency loss of 32.42% due to the rise in solar cell surface temperature*^6^. Vasisht et. al^7^. assessed the change in the efficiency of a grid-interactive rooftop PV system due to temperature variation for a year. They studied the variation in the system efficiency for different seasons of the year. For summer, efficiency decreases by 0.08% on one degree increase in PV panel temperature when average temperature of module for season (T_mod_(M)) > 45 °C. For monsoon, when T_mod_(M) > 35 °C, efficiency reduces by 0.04% on one degree increase of PV panel temperature. For post monsoon period, when T_mod_(M) > 38 °C, efficiency of module decreases by 0.06% on one degree increase of panel temperature. However, in winters, T_mod_ is 55 °C but lowest reduction in panel efficiency was observed due to reduction in ambient temperatures and cool breeze^7^.

Soiling is a major contributor to the degradation of solar photovoltaic system performance. Under on-field operation, dust particles deposit on the module’s surface and prevent solar radiation from reaching the solar cell. Solid particles with a diameter of less than 500 μm are referred to as dust^8^. Natural processes such as wind and volcanic eruptions can raise soil dust into the air, and occasionally dust may also contain minute amounts of pollen grains, hair from people and animals, fabric, and paper fibres, among other substances. Many researchers suggest that photovoltaic panels show a considerable loss in efficiency due to dust accumulation, which increases with the increasing concentration of dust^9^.

Kimber. et al.^10^ described the impact of soiling on energy output in large grid-connected SPV systems across the United States. These systems are located in the regions with little rainfall, such as the Central Valley, Northern and Southern California, and the US Desert Southwest. Soiling losses might reach 6% per year and even 20% per hour, according to the authors’ calculations. The authors also discovered that measured PV efficiencies decrease due to soiling without rainfall by 0.1% to 0.3% per day, with a mean soiling rate of 0.2% per day in the regions dry conditions. Siddiqui and Bajpai (2012) found a significant loss in panel performance and developed an equation for obtaining the soiling impact on the panel efficiency in composite climate, and further developed a co-relation between differences in efficiencies of modules with respect to the thickness of deposited panel dust^11^. Another study was performed in the state of Minas Gerais, Brazil aiming to quantify the soiling impact on PV plant performance installed in a soccer stadium. The PV power plant is composed of 5910 multi-crystalline PV modules. According to their analysis, soiling decreased rated power by roughly 13.7% during a dry period and 6.5% during a wet period. During the dry season, the energy production decreased by around 16.5%, and following the rain, it decreased by about 8.0%^12^. An experimental analysis revealed that soiling also raises module temperatures. On specific days, the daily average power reduction due to temperature rise was 0.61%, 1.04%, and 1.31%, with maximum reductions of 1.55%, 2.53%, and 3.46%, respectively. The study emphasizes the need of managing soiling-induced temperature changes, and the need for precise analysis^13^. *Sengupta et al. (2020) modeled dust accumulation on PV modules via dry deposition for particles with different diameters and modules at various tilt angles. The model shows that transmittance becomes minimum at a tilt angle of about 15° and reveals that deposition velocity depends strongly on local meteorology*^14^. *The subsequent study extended this framework by incorporating the effects of relative humidity and precipitation into dust accumulation models. Results showed that higher RH significantly increases deposition velocity while reducing rebound and resuspension*,* with particle adhesion rising nearly 80% as RH increased from 40% to 80%. Accumulated mass of PM₂.₅ and PM₁₀ grew substantially at 95% RH compared to dry conditions. Rainfall was modelled as a natural cleaning process*,* with heavy rain removing up to 96% of deposited dust*^15^. *The study was subsequently expanded by validating a physics-based soiling model across four PV plants of different capacities and locations in India*,* ranging from 40 kWp rooftop to 500 kWp canal-top systems. Results revealed generation losses up to 32.2% on certain days without cleaning*,* which reduced to 8.15% after cleaning for nearly identical irradiance conditions.*^16^.

The literature review suggests that while extensive research has been done to explore temperature-induced efficiency losses and soiling losses separately, thereby leaving, significant gap. Existing studies predominantly focused on arid or temperate climates^17–19^, lesser number of studies were performed in composite climates where high ambient temperatures and rapid dust accumulation coexist, as seen in regions like North India. Furthermore, prior work often isolates temperature and soiling effects, failing to account for their combined influence on real-world rooftop PV systems.

Addressing these gaps, this study presents several novel contributions. It offers the first empirical assessment of a 500 W_p_ grid-connected Rooftop Photovoltaic (RTPV) system under the composite climatic conditions of Lucknow, India, characterized by extreme temperatures and rapid dust accumulation. The research employs an integrated methodology that combines on-field data collected at five-minute intervals, enabling a concurrent evaluation of PV system performance under real operating conditions. The novelty of this work lies in (i) simultaneously quantifying the effects of elevated temperature, daily soiling accumulation, and wind-driven convective cooling on PV performance, (ii) evaluating the restorative effect of natural rainfall on both dust removal and thermal reduction, and (iii) comparing rooftop and simulated ground-mounted systems to highlight the thermal advantage of elevated installations. The investigation is limited to a pilot-scale installation monitored over a one-month period, chosen to capture high-resolution variations under peak summer conditions. The findings are intended to offer scalable insights relevant to small- to medium-scale rooftop PV deployments in similar climates. The outcomes are also expected to guide to deciding optimum cleaning frequency, and policy frameworks for rooftop solar deployment in urban environments.

## Experimental methodology

For the experimental analysis, a 500 Wp grid-integrated rooftop solar PV system was installed on the rooftop of the building of Institute of New and Renewable Energy, University of Lucknow, Lucknow, India (26.5° N, 81.9° E). The array was mounted on a galvanized iron (GI) structure, securely fastened to the rooftop using corrosion-resistant fasteners, bolts and brackets. The GI frame was fixed to a reinforced concrete foundation, designed using Indian Standard IS 875 (Part 3):2015 to withstand dynamic loads and prevent uplift. The design minimized mechanical vibrations and maintained positional stability under composite climatic stresses like monsoonal winds and thermal expansion. The solar PV system has 2 polycrystalline silicon panels connected in series. Both PV panels are facing toward the true south at an inclination of 26°﻿. Panels have the same electrical and mechanical properties. All panels were tested under Standard Test Condition (STC) at 1 kW/m^2^ solar radiation, 25 °C cell temperature, and 1.5 air mass. The specifications related to the electrical and mechanical properties of these panels have been listed in Table 1.

**Table 1:** Electrical and mechanical specifications of the RTPV and ground-mounted PV modules.

Parameter	Rooftop PV modules	Ground-mounted PV modules
Rated Power (Pmp)	250 Wp	250 Wp
Voltage at Maximum Power (Vmp)	34.85 V	34.85 V
Current at Maximum Power (Imp)	7.19 A	7.19 A
Open Circuit Voltage (Voc)	42.91 V	42.91 V
Short Circuit Current (Isc)	7.85 A	7.85 A
Module Efficiency	13.0%	13.0%
Temperature Coefficient of Voc	−0.34%/°C	−0.34%/°C
Temperature Coefficient of Pmp	−0.43%/°C	−0.43%/°C
PV Module Dimensions (L×W×H)	1.96 m × 0.98 m × 0.04 m	1.96 m × 0.98 m × 0.04 m
Power Output Tolerance	± 3%	± 3%

**Fig. 1:**
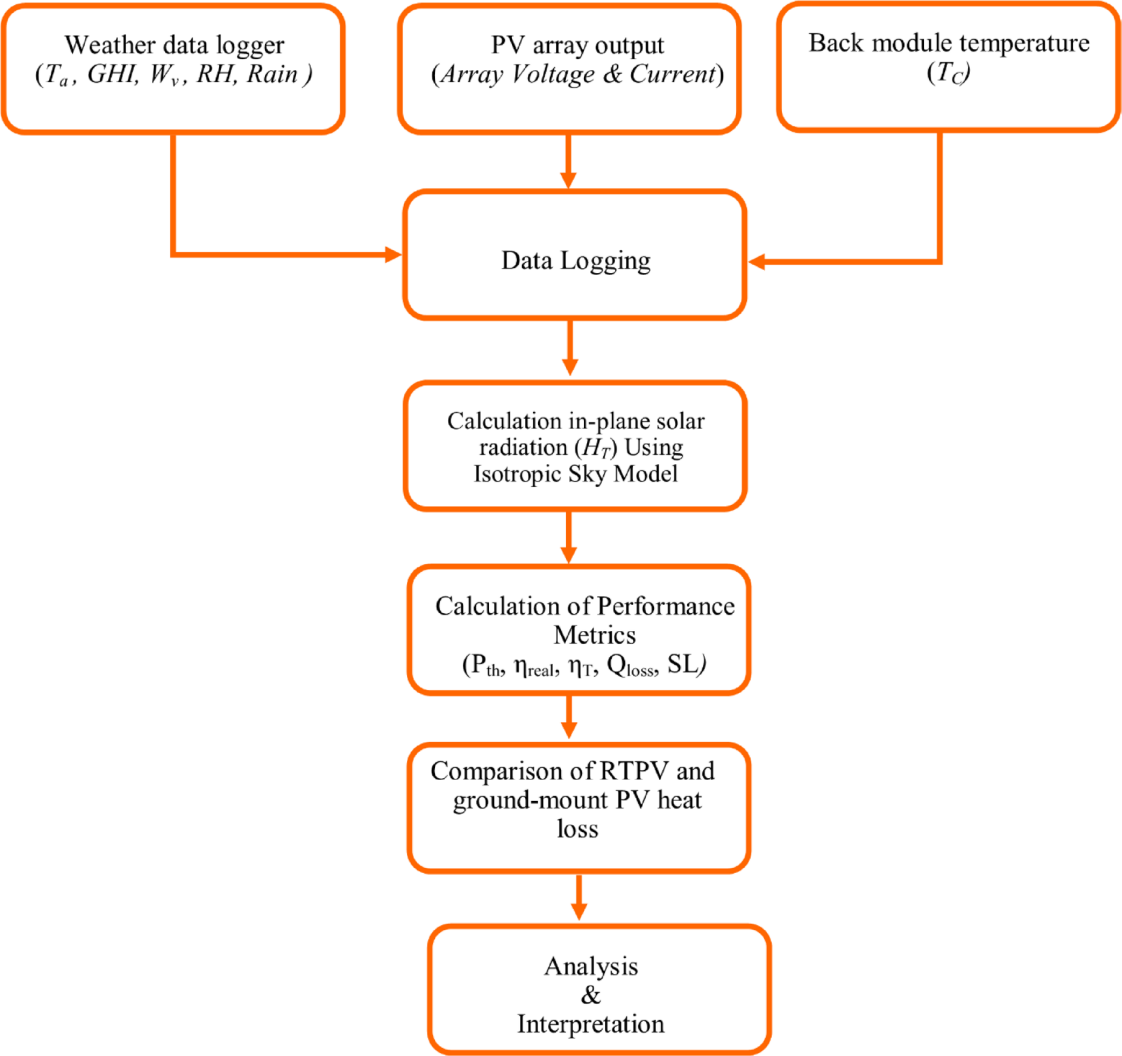
Flow chart representing the data acquisition, modeling, and performance evaluation.

The present study analyzes the performance metrics discussed in Sect. “Effect of elevated temperature on PV performance” and “Estimation of convective heat loss and wind profile adjustment” to quantify the effect of elevated temperature and soiling. The parameters were calculated with the help weather parameters, PV output, and module temperature (Fig. 1). The present study was carried out in June, representing peak summer conditions. Solar irradiance and ambient temperature (T_a_) were recorded using a weather data logger. The weather data logger also records humidity, wind speed, wind direction, and rain data at every 5-minute interval. The weather data logger is installed on the rooftop at a horizontal distance of approximately 2 m from the PV modules to accurately capture the wind velocity experienced by the system. The solar cell temperature (T_c_) is measured by using a k-type probe based digital thermometer. The thermocouple probe was firmly attached to the back surface of the module near the cell position, following standard PV monitoring practice. While back-surface values may differ slightly, prior studies show deviations within ± 2–3 °C, making this a reliable measure for operating cell temperature^20,21^. A data logger is directly connected with the PV array, which records and stores current and voltage values. The details of the equipment used and the measurement uncertainties are given in Table 2.

**Table 2:** Specification of weather data logger.

Parameter	Instrument Used	Uncertainty
Global horizontal Irradiance (GHI)	EKO Pyranometer (MS-60)	±0.8%
Air Temperature	T-type Thermocouple	± 0.5 °C
Module temperature	k-type Thermocouple	± 0.5 °C
Wind speed	Davis Instruments Anemometer (Model 6410)	±1.8 m/s
Humidity Data logger	Davis Instruments (SHT) sensor Unilog Pro (PPI) ~ 16 Ch.	±3% ± 0.25- ±0.5

### Uncertainty propagation analysis

Uncertainty propagation analysis was performed to calculate the impact of measurement uncertainties on the performance parameters of the solar PV system. The following formula has been used to calculate the uncertainty propagation:

1 $$\:{\sigma\:}_{y}=\sqrt{{\left({\frac{\partial\:y}{\partial\:{x}_{1}}\sigma\:}_{{x}_{1}}\right)}^{2}+{\left({\frac{\partial\:y}{\partial\:{x}_{2}}\sigma\:}_{{x}_{2}}\right)}^{2}+{\left({\frac{\partial\:y}{\partial\:{x}_{3}}\sigma\:}_{{x}_{3}}\right)}^{2}+\dots\:}$$

Where

y = A parameter which relies on multiple measured quantities $\:{x}_{1}$_,_ $\:{x}_{2}$_, ……_.$\:{x}_{n}$​.

$\:{\sigma\:}_{y}$= Uncertainty in the calculated parameter.

$\:{\sigma\:}_{{x}_{i}}$= Uncertainty in measured values of $\:{x}_{i}$.

$\:\frac{\partial\:y}{\partial\:{x}_{i}}$ = The partial derivative of y with respect to $\:{x}_{i}$.

The uncertainty in theoretical power output P_th_, calculated using the corrected model accounting for irradiance and temperature, was found to be ± 0.838%. This moderate uncertainty results from combined measurement errors in irradiance and module temperature. The temperature-corrected efficiency η(T) showed a minimal uncertainty of ± 0.028%. The real-time efficiency η_real_ had an uncertainty of ± 0.94%, primarily influenced by power and irradiance measurement accuracy. Convective heat loss (Q_loss_) exhibited an uncertainty of ± 7.67%, and the uncertainty in soiling loss (SL) was ± 0.93%, reflecting the impact of instrument error.

**Fig. 2:**
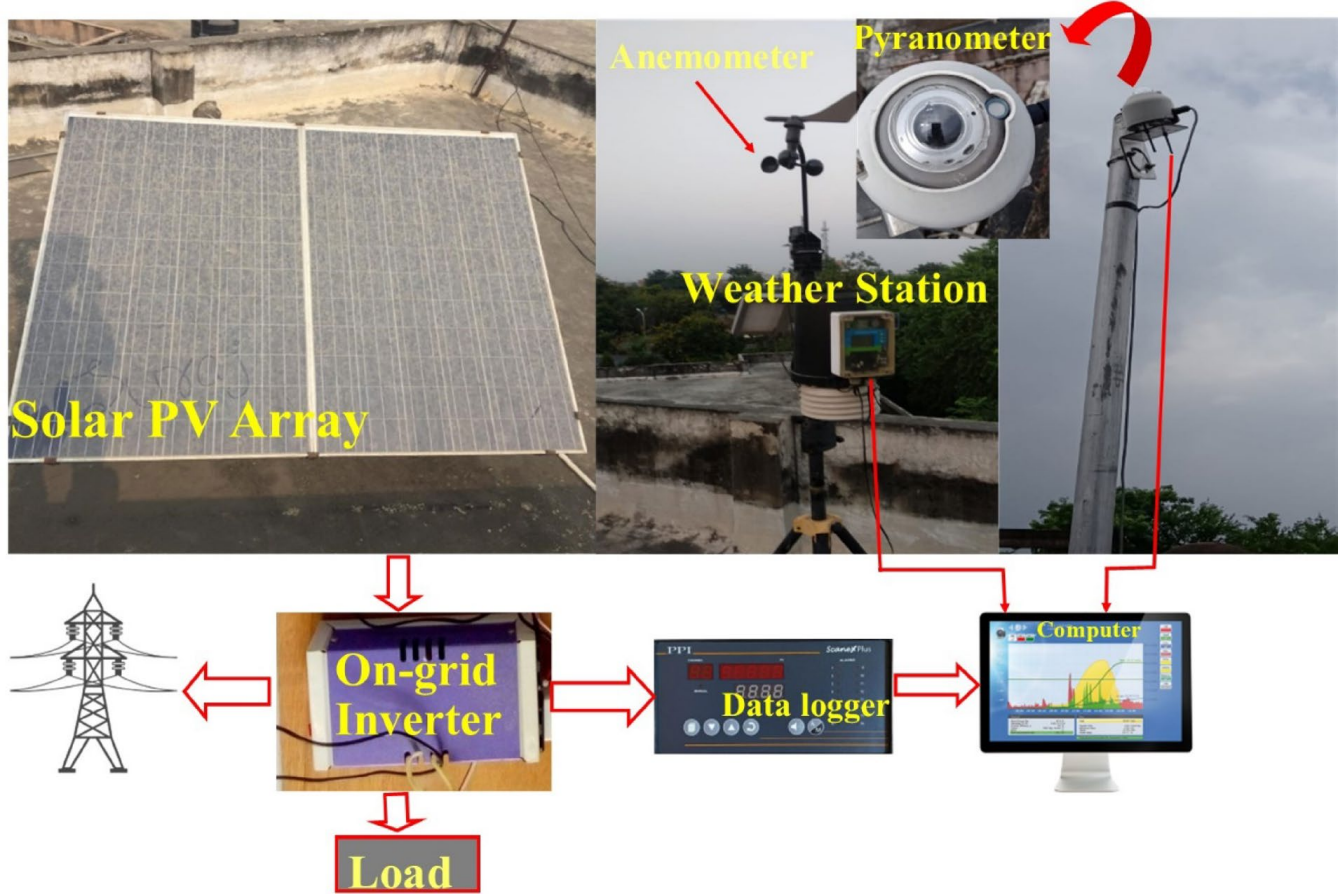
Schematic diagram of the experimental setup showing the rooftop PV array and weather station connected to a data logger for real-time monitoring.

Figure 2 illustrates the setup comprising the solar PV array, weather station, and data logging system. A 500 Wp PV array, mounted on the rooftop of a 15-meter-high building, was employed to assess the soiling effect. All collected data, including electrical and meteorological parameters, is sent to a data logger. The power generated by the PV system was fed directly into the grid. The weather station is also linked to the data logger, enabling continuous acquisition of environmental parameters. Using the recorded PV output and weather data, performance variations can be effectively analyzed. All the instruments – the K-type thermocouple, pyranometer, and data logger were pre-calibrated according to National Institute of Standards and Technology (NIST) protocols. Additionally, all sensors were shielded to prevent direct solar heating, ensuring accurate measurements.

### Radiation on solar PV plane

The weather data logger provides global horizontal irradiance (GHI) at every five-minute interval. Global horizontal insolation (H) for a day has been calculated by taking the sum of every five-minute irradiance over the day. The modules were installed at an angle of 26^o^ (latitude of the site) and insolation on the module surface was required for the output power calculation. So, insolation on the inclined surface was calculated from global horizontal insolation using the isotropic sky model, one of the most widely applied approaches due to its simplicity and reasonable accuracy. Previous studies have shown that it performs reliably in composite and moderately cloudy climates where detailed diffuse data are unavailable^22,23^. Hence, it was chosen as an appropriate method for estimating tilted-surface irradiance in rooftop PV analysis.

2 $$\:{\text{H}}_{\text{T}}={\text{R}}_{\text{b}}{\text{H}}_{\text{b}}+{\text{H}}_{\text{d}}\left(\frac{1+\text{c}\text{o}\text{s}{\upbeta\:}}{2}\right)-\text{H}{{\uprho\:}}_{\text{g}}\left(\frac{1-\text{c}\text{o}\text{s}{\upbeta\:}}{2}\right)$$

Where.

$\:{{\uprho\:}}_{\text{g}}$ = ground reflectance which is taken (0.2).

In addition, R_b_ is a geometric factor and is defined as the ratio of beam radiation on the sloped surface to beam radiation on the horizontal surface at a time, which is evaluated accurately by using the Eq. 3.

3 $$\:{\text{R}}_{\text{b}}=\frac{\text{cos}\left({\varnothing}-{\upbeta\:}\right)\text{cos}{\updelta\:}\:\text{c}\text{o}\text{s}{\upomega\:}+\text{sin}\left({\varnothing}-{\upbeta\:}\right)\text{s}\text{i}\text{n}{\updelta\:}}{\text{c}\text{o}\text{s}{\varnothing}\text{c}\text{o}\text{s}{\updelta\:}\text{c}\text{o}\text{s}{\upomega\:}+\text{s}\text{i}\text{n}{\varnothing}\text{s}\text{i}\text{n}{\updelta\:}}$$

H_b_ is beam radiation and can calculate by subtracting diffuse radiation from total radiation.

4 $$\:{H}_{b}=H-{H}_{d}$$

above expression is used to calculate the daily diffuse radiation (H_d_) from daily global radiation (H) and sky clearness index (K_T_) value^23^.

For $$\:{{\upomega\:}}_{\text{s}}\le\:81.4^\circ\:$$

5 $$\:\frac{{\text{H}}_{\text{d}}}{\text{H}}=\left\{\begin{array}{c}1.0-\:\\\:0.143\end{array}\genfrac{}{}{0pt}{}{0.2727{\text{K}}_{\text{T}}+2.4495{\text{K}}_{\text{T}}^{2}-11.9514{\text{K}}_{\text{T}}^{3}+9.3879{\text{K}}_{\text{T}\:\:}^{4}\:\:}{.}\genfrac{}{}{0pt}{}{\text{f}\text{o}\text{r}\:{\text{K}}_{\text{T}}<0.715}{\text{f}\text{o}\text{r}{\:\text{K}}_{\text{T}}\ge\:0.715}\right.$$

And for $$\:{{\upomega\:}}_{\text{s}}>81.4^\circ\:$$

6 $$\:\frac{{\text{H}}_{\text{d}}}{\text{H}}=\left\{\begin{array}{c}1.0+\:\\\:0.175\end{array}\genfrac{}{}{0pt}{}{0.2832{\text{K}}_{\text{T}}-2.5557{\text{K}}_{\text{T}}^{2}+0.8448{\text{K}}_{\text{T}}^{3}\:\:\:\:\:\:}{.}\genfrac{}{}{0pt}{}{\text{f}\text{o}\text{r}\:{\text{K}}_{\text{T}}<0.722}{\text{f}\text{o}\text{r}{\:\text{K}}_{\text{T}}\ge\:0.722}\right.$$

*In equation*,* the term* $\:\left(\frac{{H}_{d}}{H}\right)$ *represents the* diffuse fraction. *This ratio quantifies the proportion of diffuse radiation relative to the total global radiation and is essential for accurately estimating the radiation incident on an inclined surface.* The value of K_T_ depends upon atmospheric clearness, which gives the value of diffuse radiation. It is evaluated from the ratio of global and extraterrestrial radiation on horizontal surface.

7 $$\:{{\text{K}}_{\text{T}}=\frac{\text{H}}{\text{H}}}_{0}$$

Where:

H = Daily average hourly radiation on horizontal surface (kWh/m^2^/day), and H_o_ = Daily average hourly extraterrestrial radiation on horizontal surface (kWh/m^2^/day).

### Effect of elevated temperature on PV performance

Solar cell yield is affected by solar irradiance and ambient temperature. The power of a SPV module under the influence of temperature and irradiance was calculated as^24^:

8 $$\:{P}_{th}={P}_{STC}\times\:\frac{{G}_{T}}{{G}_{STC}}\times\:\left[1-\alpha\:\left({T}_{C}-{T}_{STC}\right)\right]$$

Where $\:{P}_{th}$ is theoretical power of the PV modules, T_*C*_ = cell temperature (^o^C), $\:\alpha\:$ = temperature coefficient of power (%/^o^C), and T_STC_ is the temperature at standard test condition taken as 25 °C during calculation. This model provides the theoretical PV output based on measured irradiance and cell temperature, allowing us to quantify thermal derating effects on module performance. To account for temperature effects, the percentage power loss relative to Standard Test Conditions (STC) is calculated using the standard linear temperature correction^20^:

9 $$\:{P}_{loss}\left(\%\right)=\alpha\:\left(\varDelta\:T\right)$$

This relation directly quantifies the impact of elevated operating temperature on output power.

Similarly, the temperature-corrected efficiency calculated using following formula^25^:

10 $$\:\eta\:\left(T\right)={\eta\:}_{ref}[1+\alpha\:({T}_{C}-{T}_{STC}\left)\right]$$

Where: η_ref​_ = PV module efficiency at STC.

The on-field efficiency of a SPV modules is calculated using the following formula^26^:

11 $$\:{\eta\:}_{real}=\frac{{P}_{real}}{{A}_{m}\times\:{G}_{T}}\times\:100$$

Where P_real_ is the recorded output power of the array, G_T_ is the plane of array irradiance (W/m^2^) and *A*_*m*_  is the area of the SPV module (m^2^).

### Estimation of convective heat loss and wind profile adjustment

To analyze the effect of elevated temperature on the PV output, an empirical equation adapted from the PVUSA model, has been incorporated to compute the heat loss by convection (Q_loss_)^27^. It accurately represents convective cooling losses in rooftop PV systems exposed to variable wind speeds. It directly incorporates wind velocity and temperature differences, making it appropriate for current site-specific analysis.

12 $$\:{Q}_{loss}={U}_{c}+\left({U}_{v}\cdot\:{W}_{v}\right)\cdot\:{A}_{m}\cdot\:({T}_{c}-{T}_{a})$$

Where: U_c_ = Constant heat loss factor due to natural convection (W/m^2^K), U_v_ = Wind dependent heat loss factor (W/m^2^K.m/s), $\:{W}_{v}$ = Wind velocity (m/s), A_m_ = Area of the module (m^2^), T_c_ = Cell temperature (^o^C), T_a_ = Ambient temperature (^o^C). The U_c_ represents wind loss factor with an empirical value proposed by PVUSA NREL for the calculation of heat loss due to the corresponding wind speeds.

The equation is used to estimate convective heat loss from rooftop PV modules because it accurately represents real-world heat dissipation under varying wind and temperature conditions. The temperature difference between the module surface and ambient air drives the convective heat transfer. Measured parameters like ambient temperature, cell temperature, wind velocity and module’s dimensional parameters were employed to calculate the real heat loss values. In rooftop systems, modules are typically more exposed to atmospheric airflow, making wind-induced convection a major factor in cooling which displays better performance compared to the ground mount modules^28^. The real heat loss values of RTPV were also compared with a simulated ground-mounted SPV system at 2-meter height. Simulated PV system has similar technical characteristics with rooftop SPV system. To calculate the wind velocity at 2-meter height we used vertical wind profiles over urban areas by choosing the roughness length z_o_ characterizes the terrain and is critical for accurate calculations.

13 $$\:v\left(h\right)={v}_{o}\times\:\frac{ln\left(h/{z}_{o}\right)}{ln\left({h}_{o}/{z}_{o}\right)}$$

Where: $\:{v}_{o}\:$= wind speed at reference height h_o_, h = height at which wind speed calculated, h_o_ = reference height (at which $\:{v}_{o}$​ is known), z_o_ = roughness length. The required wind data has been taken from the weather station located on the roof to the building.

Long-term wind statistics were obtained from the MERRA-2 (Modern-Era Retrospective analysis for Research and Applications, Version 2) database—a NASA global reanalysis dataset that assimilates satellite and surface observations to ensure the representativeness of the observed data^29^. It provides high-resolution atmospheric and surface parameters over extended timeframes and is widely used in climate, renewable energy, and environmental studies.

### Soiling loss Estimation

The soiling effect has been determined with the help of daily recorded PV module output power. It was observed that the soiling density varies from the start to the end of the month, influenced by weather parameters. The soling loss was calculated by using the following formula^30^:

14 $$\:Soiling\:loss\:\left(SL\right)\left(\%\right)=\left(1-\frac{{P}_{real}}{{P}_{th}}\right)\times\:100$$

Where P_th_ is theoretical output power of the SPV module power as discussed in Eq. 8, P_real_ is its real output power recorded during the study. The equation estimates daily soiling losses by comparing corrected theoretical power (temperature- and irradiance-adjusted) with real measured output.

*To evaluate how soiling accumulates over time*,* the rate of soiling loss (r*_*SL*_*) was computed as the difference in daily soiling loss between two consecutive dry days*:

15 $$\:{r}_{SL}={SL}_{d}-{SL}_{(d-1)}$$

*expressed in units of %/day. Here*,* SL*_*d*_ *and SL*_*(d−1)*_ *represent the average daily soiling loss on day ‘d’ and day ‘d−1’*,* respectively. After rainfall events (which act as natural cleaning)*,* the SL value was reset to a new baseline*,* and* $\:{r}_{SL}$ *was recalculated from that point onward.*

## Results and discussion

**Fig. 3:**
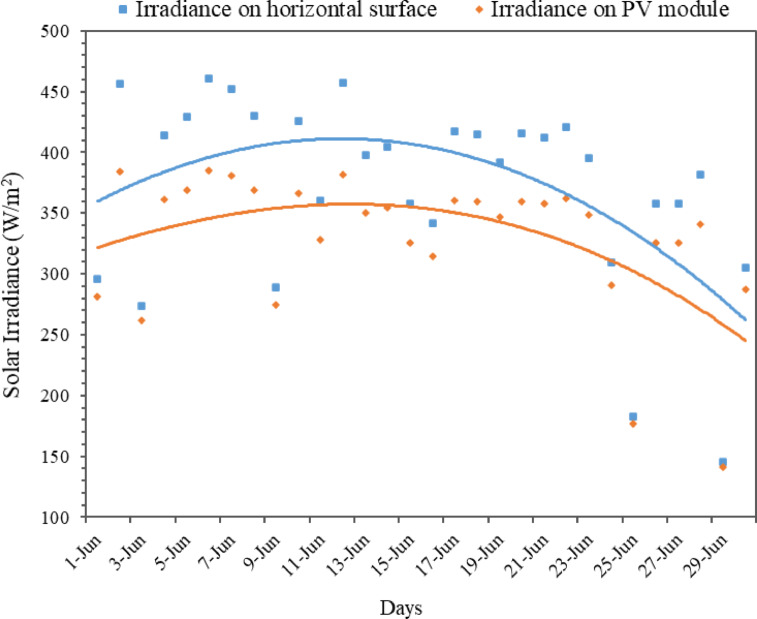
Daily global solar irradiance on the horizontal surface and plane of array for June.

Figure 3 shows the daily irradiance on the horizontal surface and on the PV module plane for the June month. Irradiance on horizontal surface was measured using a pyranometer and then converted to represent irradiance on the PV module using the Isotropic Sky Model. This model considers diffuse sky radiation, simulating the amount of sunlight that reaches a tilted PV surface under typical sky conditions. The data has some small variations between the recorded irradiance on horizontal surface and calculated irradiance on PV module surface. The horizontal irradiance ranges from a minimum of 145 W/m² (on June 29) to a maximum of 461 W/m² (on June 6), and the plane of array irradiance ranges from 141 W/m² to 385 W/m² on the same respective days. These fluctuations are due to varying weather conditions like cloud cover and rain. The monthly average irradiance on the horizontal surface was approximately 371 W/m², and the monthly average value for plane of array irradiance was around 334 W/m².

**Fig. 4:**
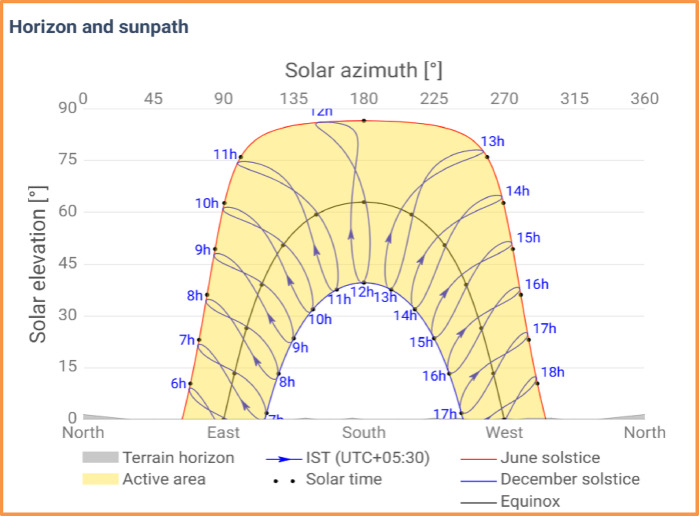
The horizon and sun path diagram of the site over the year, showing solar exposure from summer solstice to winter solstice.

Figure 4 represents the sunpath diagram of the site over the year. The figure shows the active solar area PV system receives from summer solstice, to winter solstice. Gray line highlights horizon suggesting during the whole year there is no shadow casting by obstacles or self-shading. This figure suggests that the site provides the optimum condition for the operation of solar PV system, ensuring maximize solar exposure and power production throughout the year. So, the sun path diagram suggests that the horizon shading effect for the site is nil and is confined to the 180^o^ south.

### Effect of wind on PV module heating

**Fig. 5:**
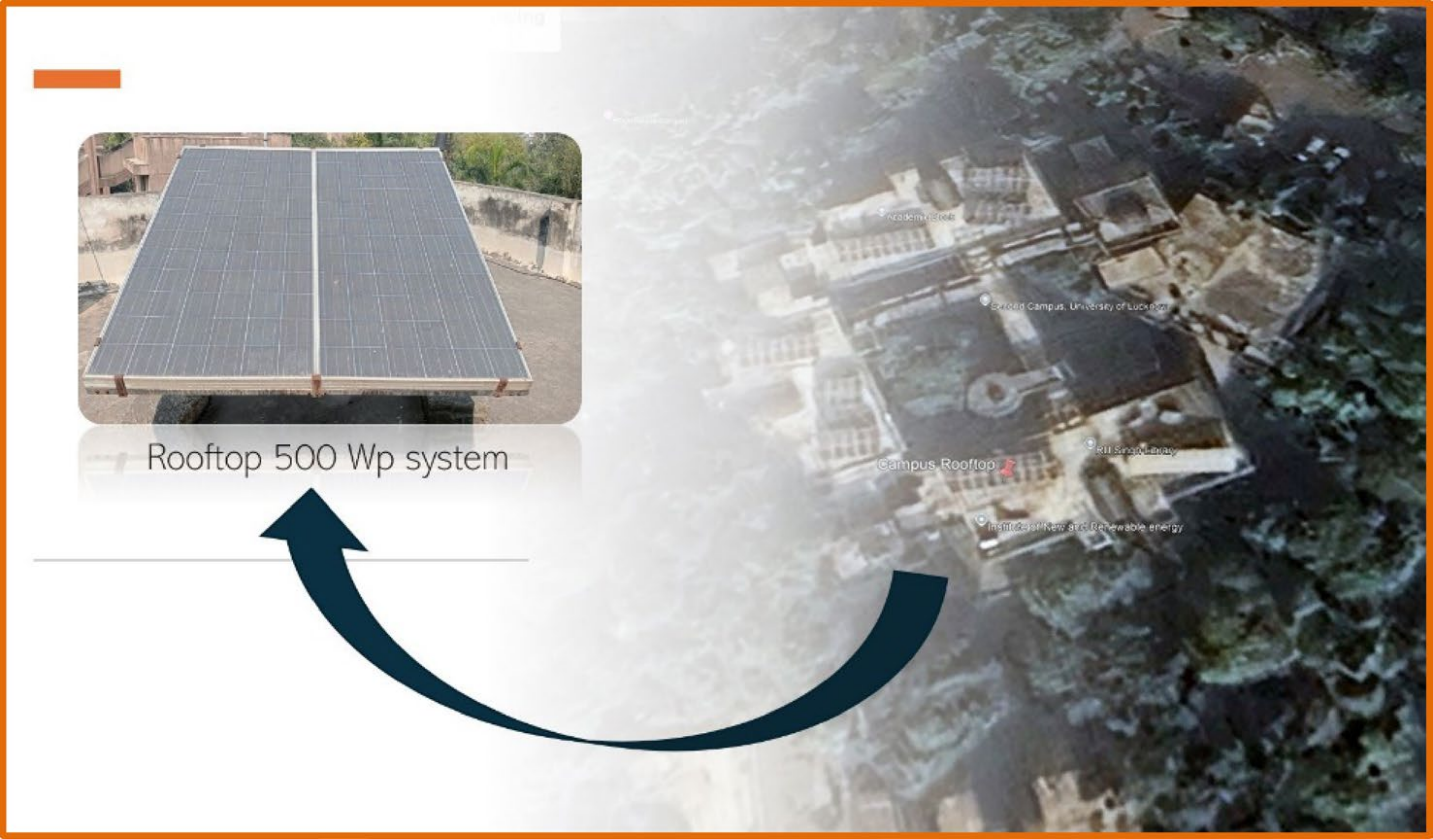
Rooftop PV system (500 Wp) installed on the institute’s building illustrating surrounding environment, including nearby buildings and vegetation. Figure is created by using Google Earth (https://earth.google.com/web).

Wind is an important weather parameter which can reduce the PV module heating^31^. So, the exposure of PV array to the wind is important to maximize the convective heat transfer. In the present study, the rooftop-mounted PV modules are installed on a rooftop at a height of approximately 15 m. This elevated placement, combined with relatively open surroundings—including only a few nearby buildings, sparse shrubs, and isolated tall trees—minimizes the impact of terrain-induced surface roughness on wind flow. As a result, the modules benefit from more consistent and unobstructed wind exposure. The positioning allows the free-flowing air to sweep across the module surfaces effectively, thereby enhancing convective cooling. This favourable site along with the PV system’s surroundings and its exposure to natural wind flow are depicted in Fig. 5.

**Fig. 6:**
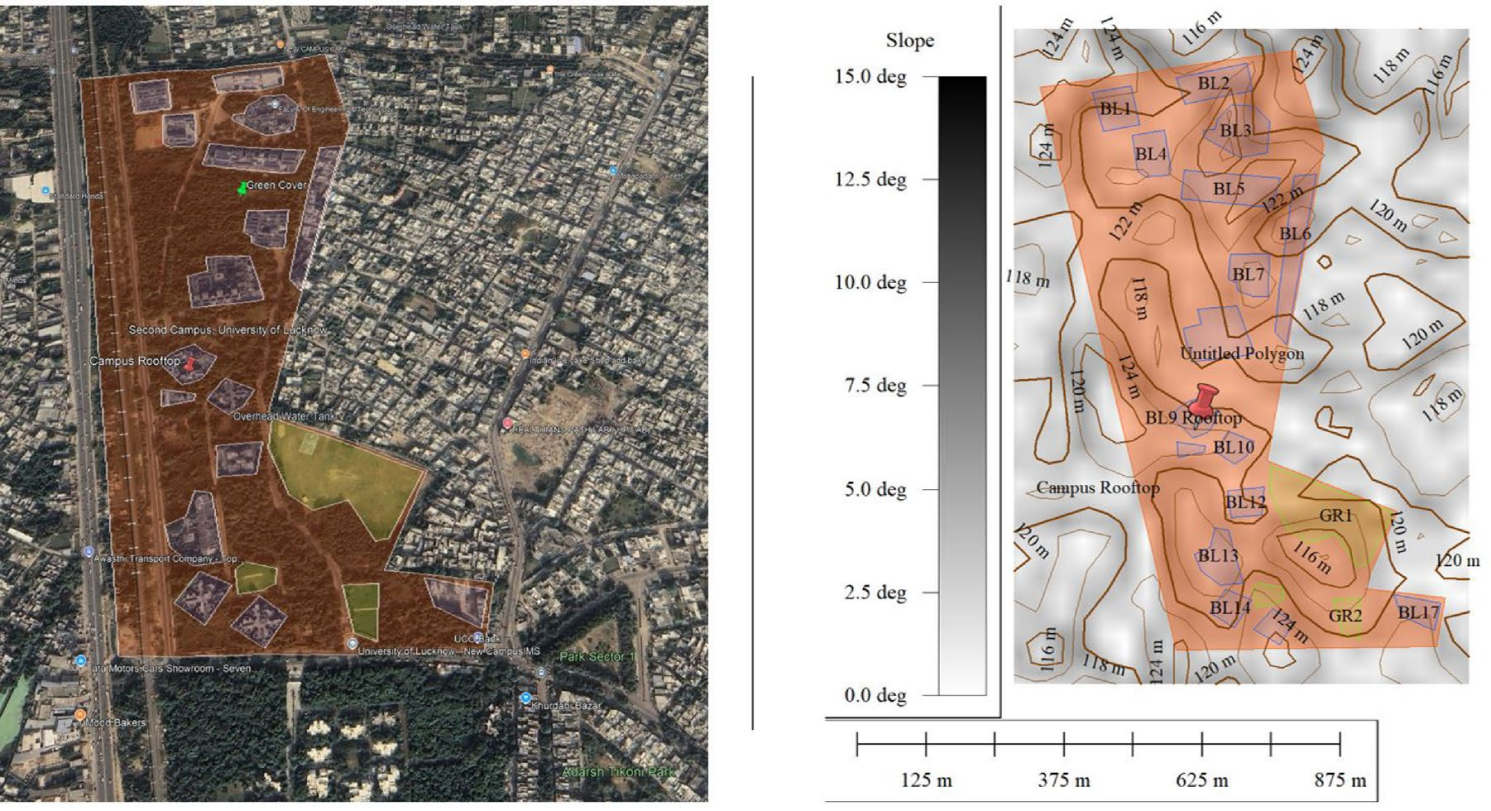
Location of the campus with green canopy surrounded by the concrete structures. The study area map is created using geographic information system (GIS) software (Global Mapper v26.1; version 26.1 © Blue Marble Geographics).

Figure 6 displays the wind velocity profile assuming different heights of the building, providing information about daily and annual fluctuations. Which is important for evaluating shading effects because larger obstructions can hinder the velocity for long periods. The picture also depicts peak wind speeds, which affect PV module stability and pose possible structural hazards. Wind speed measurement data helps in optimizing PV panel installation by reducing shade losses and understanding soiling trends. Consistent airflow promotes passive cooling and increases energy output; however, high wind speeds may demand structural reinforcements.

Table 3 highlights the wind characteristics at the site, such as average wind velocity and roughness length. The average wind speed measured at the rooftop height of 15 m was 8.9 m/s, indicating strong wind exposure that facilitates effective convective cooling of the PV modules and helps mitigate temperature-induced efficiency losses. In comparison, the estimated wind speed at 2 m above ground was 4.7 m/s, calculated using the logarithmic wind profile with a roughness length (z₀) of 0.2^32^, which is consistent with values used in recent studies of semi-urban terrain for built areas containing shrubs, buildings, and sparse vegetation^33,34^. This difference highlights the advantage of elevated PV installation, which benefits from increased wind flow and reduced surface roughness effects. The short-term variability in rooftop wind speed, represented by a standard deviation of 3.93 m/s during June, reflects significant diurnal fluctuations due to changing weather patterns, which can influence not only convective cooling but also dust accumulation and removal. From MERRA-2 dataset, the long-term standard deviation in wind velocity was found to be 0.076 m/s at 2 m and 0.132 m/s at 15 m, averaged over the 2010–2020 period. These relatively low values indicate stable wind conditions at the site over the last decade, justifying that the June 2024 wind measurements are not outliers but representative of typical rooftop wind behavior.

**Table 3:** Short-term and long-term wind velocity profiles at the site.

S No.	Wind profile	Details	Velocity (m/s)
1.	Average wind velocity at rooftop (15 m)	Measured	8.9
2.	Average wind velocity at ground (2 m)	(Log Law Z_O_ = 0.2)	4.7
3.	Wind velocity variability STDEV 15 m	June	3.93
4.	Long-term variability in Wind Velocity at 2 m	MERRA-2 (2010–2020)	0.076
5.	Long-term variability in Wind Velocity at 15 m	MERRA-2 (2010–2020)	0.132

### Variation of weather parameters

**Fig. 7:**
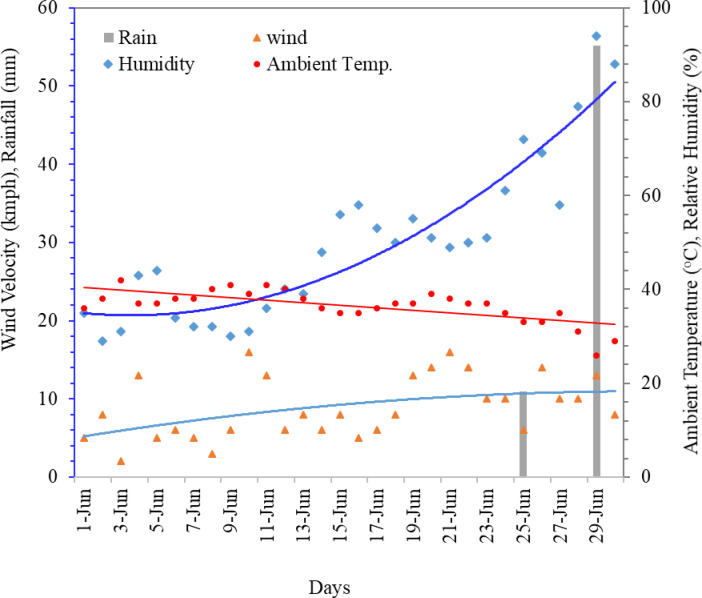
Variation of wind velocity, ambient temperature, relative humidity and rain for June. Regression lines highlight the overall trends of weather parameters providing clearer interpretation beyond short-term variability.

The weather parameters have an impact on a PV system’s performance. Temperature is important in deciding the solar PV system net energy loss. Depending on the variation of climatic parameters like solar radiation, ambient temperature, soiling, and wind speed, the temperature of solar PV modules also varies. For polycrystalline silicon PV modules, the optimal operating temperature range is generally close to the Standard Test Condition (STC) reference of 25 °C, with efficiency remaining relatively stable up to ~ 30 °C. Beyond ~ 45 °C, a noticeable decline in efficiency occurs, and performance degradation becomes significant above 60 °C, where voltage drop dominates output power losses. In our study, peak cell temperatures reached 64 °C, aligning with this critical derating threshold. Figure 7 displays the change in ambient temperature, wind speed, relative humidity, and rain. In June, the maximum daily average hourly relative humidity was recorded to be 94.0% and the minimum humidity was 29.0%. Good wind speeds promote the convective heat loss of the module that keeps its temperature down and avoids power reduction^35^. The maximum daily mean hourly wind velocity was measured at 16 km/h on June 10 and 21, while the minimum velocity was 2 km/h on June 3. Rain acts like an anti-soiling agent, removing the deposited dust from the PV panels and keep their temperature down. The maximum amount of rain, 55.1 mm, was logged on June 29. On June 21 st, a reduction of 15 °C in cell temperature was measured compared to the average daily temperature of the month.

### Effect of ambient temperature and radiation on cell temperature

Compared to ambient temperature, a solar photovoltaic module temperature rises quickly. A solar PV module cannot convert the complete spectrum of solar radiation into electric energy. The photonic energy equivalent of the bandgap (∆Eg) of the solar cell gets converted, while the excess radiation acts as a heating component for the module in addition to the ambient temperature. The dark color of the solar cell and the vacant space between the solar cell and encapsulation material contribute to the enhanced temperatures of module. Both solar insolation and temperature parameters can be utilized to maximize the performance of solar PV panels for potential commercial purposes^36^.

**Fig. 8:**
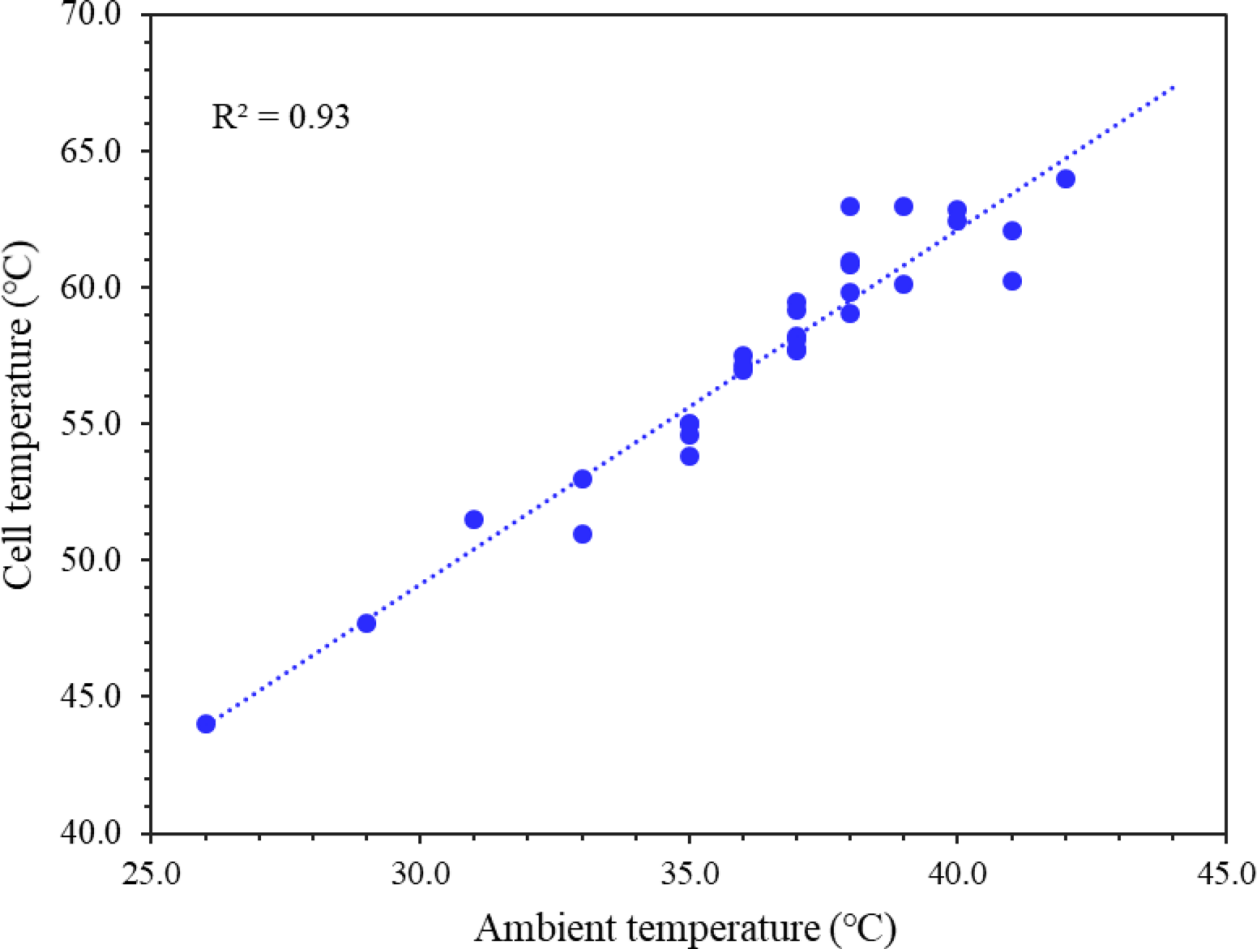
Variation of cell temperature (T_C_) and ambient temperature (T_a_) for June, showing a correlation between the two parameters.

As shown in Fig. 7, the maximum ambient temperature (T_a_) was 42.0 °C (measured on 3rd June) and the corresponding cell temperature (T_C_) was 64.0 °C (its highest value for the month). Similarly, the minimum T_a_ was found to be 26 °C (recorded on 29th June) and the corresponding T_C_ of 44.0 °C was its lowest value for the month. Due to the increased exposure of the sun’s radiation during summer, PV modules temperature rises strongly compared to other seasons. Figure 8 also shows a strong correlation (R^2^ = 0.93) between ambient temperature and cell temperature. Which suggests that solar cell temperature is directly affected by ambient temperature. Additionally, surrounding concrete surfaces and structures have a significant effect, increasing module temperature due to the heat island effect.

**Fig. 9:**
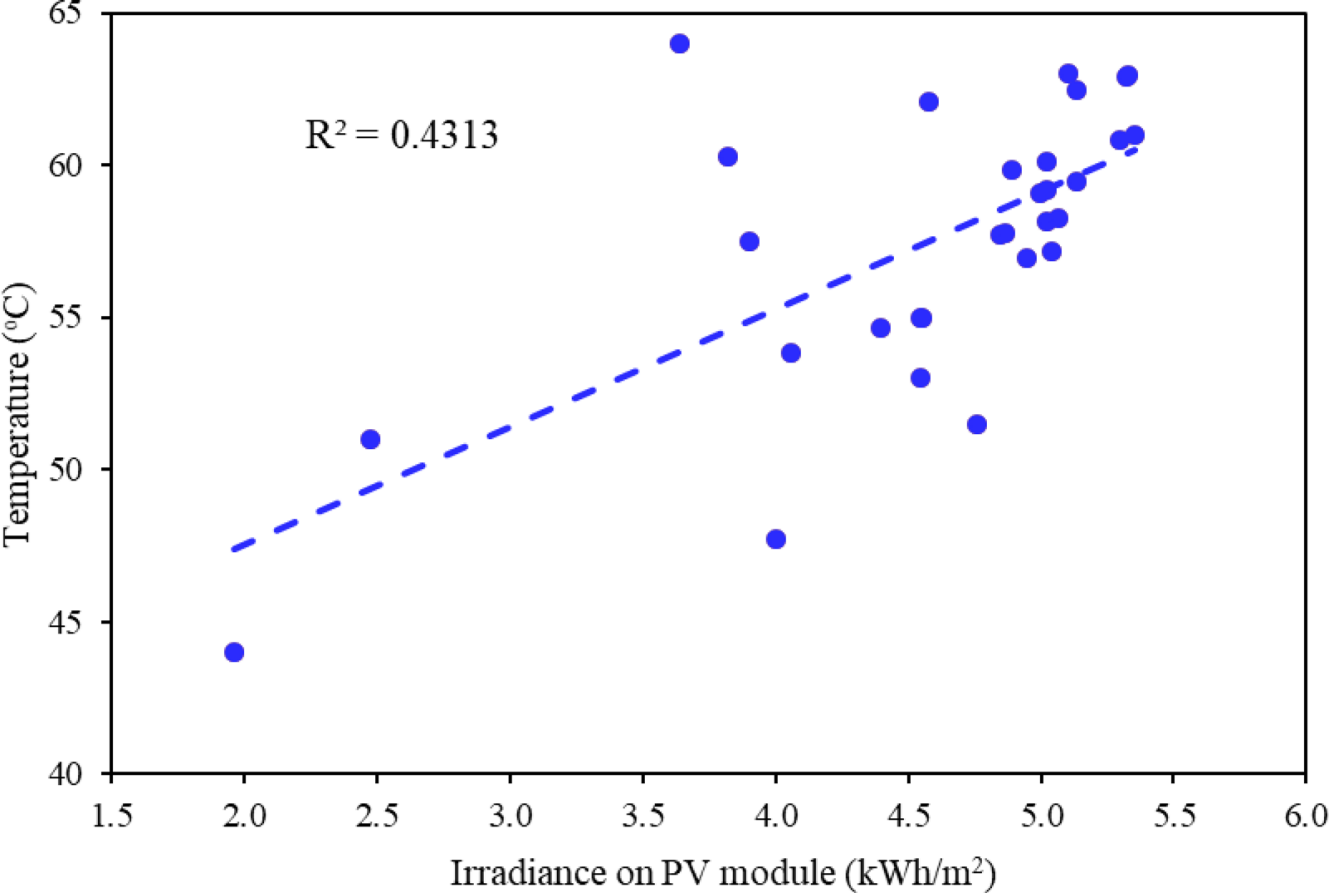
Effect of solar radiation on cell temperature of installed rooftop PV system.

Although solar energy generates PV power, it also raises the operating temperature of the solar cell. Figure 9 proves that solar radiation plays a critical role to increase the cell temperature (T_c_). In June, the highest solar insolation was registered on 6th June, while the lowest solar irradiance on 29th June. Average daily solar radiation for June was recorded as 4.5 kWh/m^2^/day. Although Fig. 9 suggests a moderate correlation (R² = 0.43) between irradiance and cell temperature, the scatter indicates that irradiance alone cannot fully explain module heating. Wind-driven convection substantially modifies this relationship by dissipating absorbed heat. For instance, as discussed in Sect. “Convective heat transfer and PV power loss”, during days with average rooftop wind speeds above 8 m/s, module temperature was reduced by about 4 °C compared to calm days of similar irradiance, confirming the cooling role of airflow. On average, rooftop modules exhibited 16.19% higher convective heat loss compared to the simulated ground-mounted case, reflecting the effect of stronger rooftop wind speed as 8.9 m/s at 15 m vs. 4.7 m/s near ground. These results demonstrate that optimal PV performance under composite climates arises not only from irradiance availability but also from sustained wind speeds (> 8 m/s), which enhance passive cooling and mitigate efficiency losses.

**Fig. 10:**
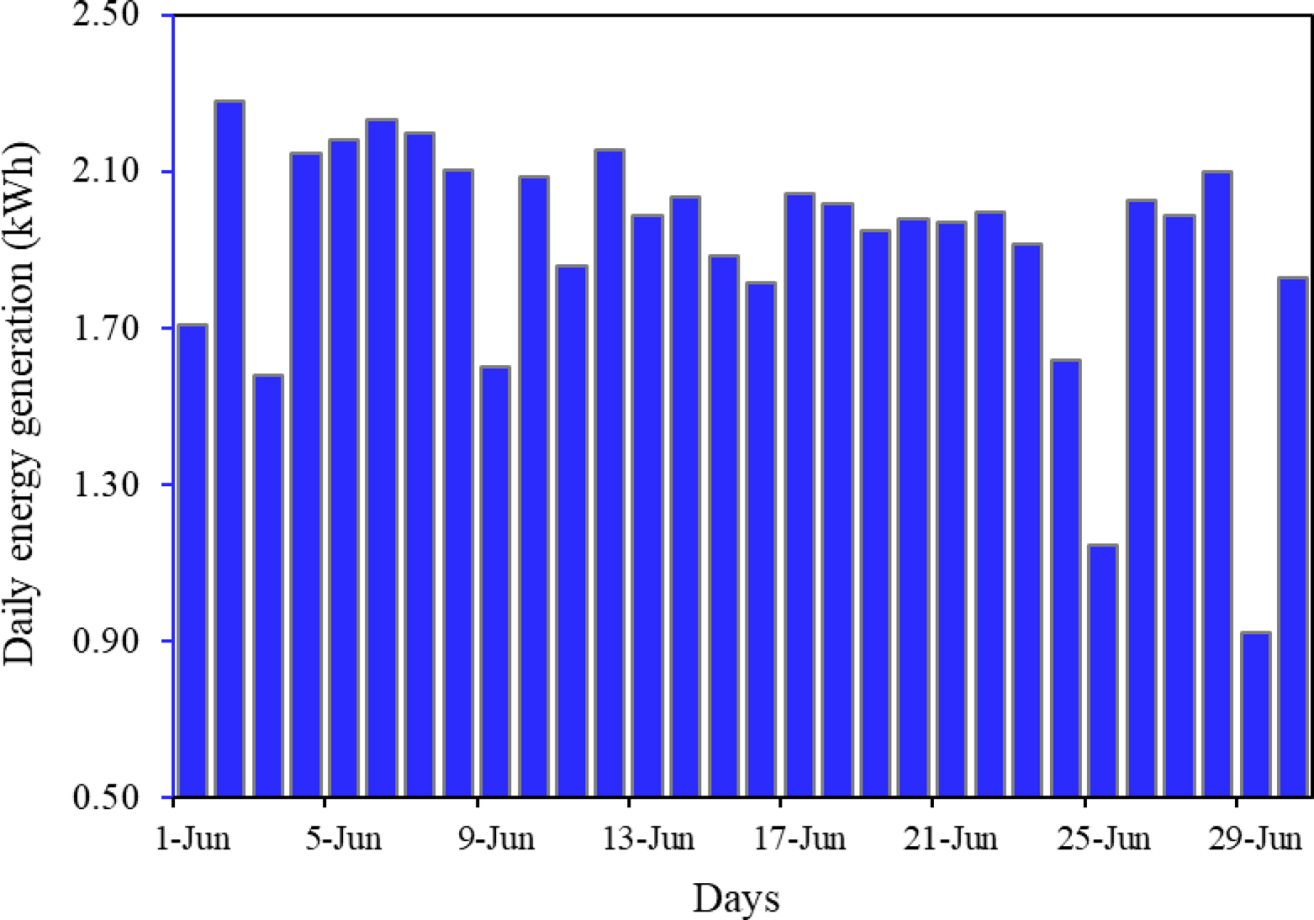
Daily and cumulative energy generation of the system over the month of June.

Figure 10 shows the daily and cumulative energy generation by the installed rooftop PV system for the June month. The figure provides insight about system’s performance under extreme weather conditions like temperature and soiling. It reveals the fluctuations in daily energy generation, ranging from a minimum value of 0.92 kWh on June 29 to a maximum of 2.28 kWh on June 2. The variation in energy generation of the PV modules is due to changes in solar radiation, cell temperature and *other climatic parameters*. Maximum energy production days was recorded on June 2 (2.28 kWh) and June 6 (2.23 kWh) while minimum on June 25 and 29 with a value of 1.15 and 0.92 kWh respectively. Cumulative energy generation shows the total of energy production from 1 st June to 30th June. Notable reduction in daily energy generation was recorded on days like June 25 (1.15 kWh) and June 29 (0.92 kWh) due to heavy rain. The figure provides insight about system’s performance under extreme weather conditions like temperature and soiling.

### Effect of temperature

**Fig. 11:**
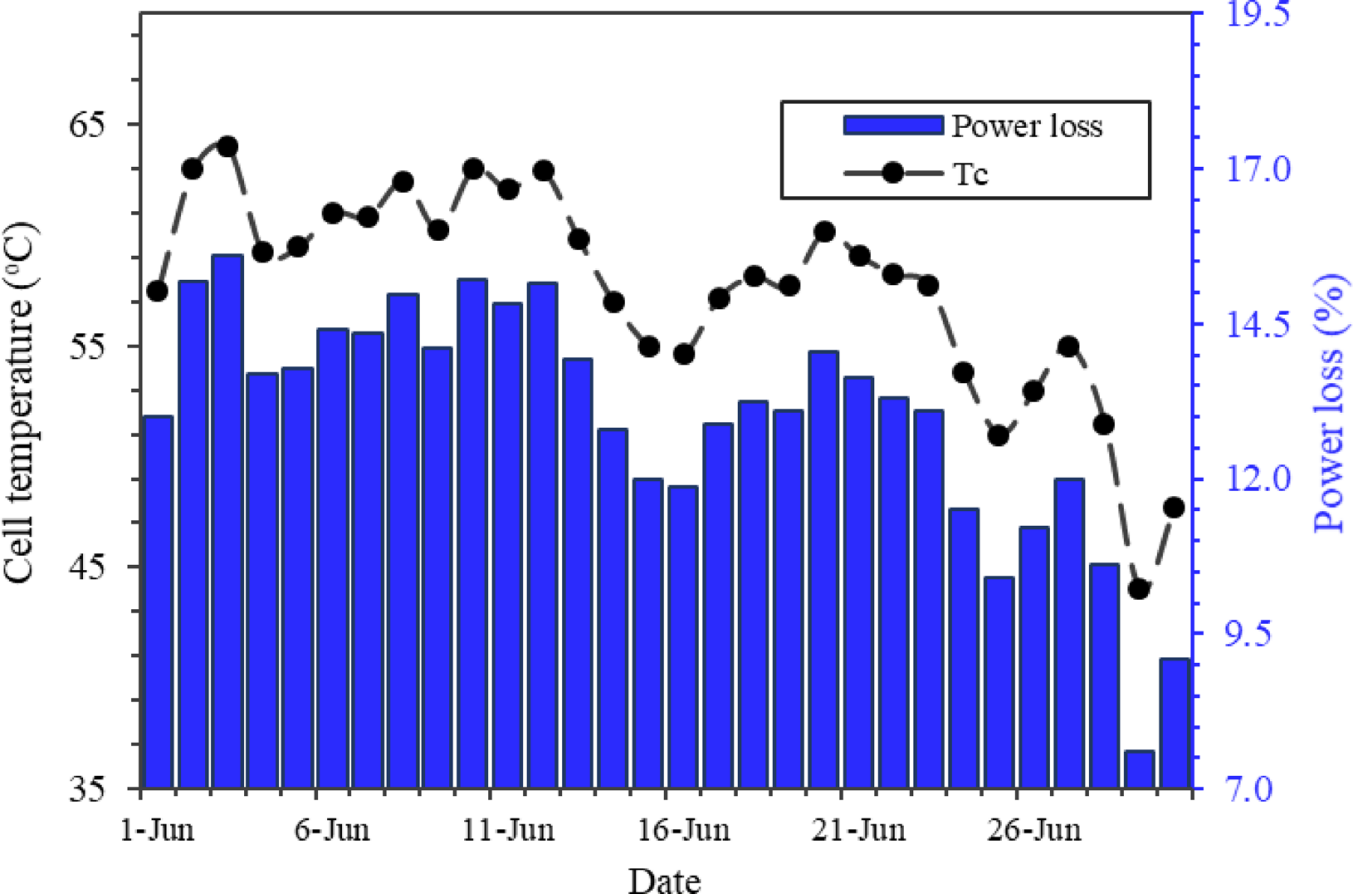
Daily power loss (%) due to elevated cell temperature for the month.

Figure 11 illustrates the variation in cell temperature (T_C_), and percentage power loss due to elevated cell temperature. A linear regression analysis further quantified the relationship between cell temperature and normalized power loss (*δP*), yielding the model:

$$\:\delta\:P=0.137\times\:{T}_{C}-1.307$$

where *δP* is the percentage power loss relative to STC, and *Tc*​ is the cell temperature (°C). This empirical model corroborates the theoretical relationship (Eq. 12) and underscores the dominant role of temperature in efficiency reduction under composite climatic conditions.

Cell temperature consistently exceeds ambient temperature because of heat absorption. Figure 11 presents the daily variation of cell temperature and the corresponding percentage power loss due to elevated temperature for the month of June. The results clearly show that power loss varies according to the cell temperature, with higher module temperatures consistently leading to larger reductions in output power. Peak cell temperatures of around 64 °C was recorded on 2nd June correspond to maximum daily power losse 15.6%, whereas lowest cell temperature of 44 °C on 29th June results in reduced loss of about 7.6%. On average, daily power loss due to elevated temperature was approximately 13.2%, confirming the strong inverse relationship between operating temperature and PV efficiency.

**Fig. 12:**
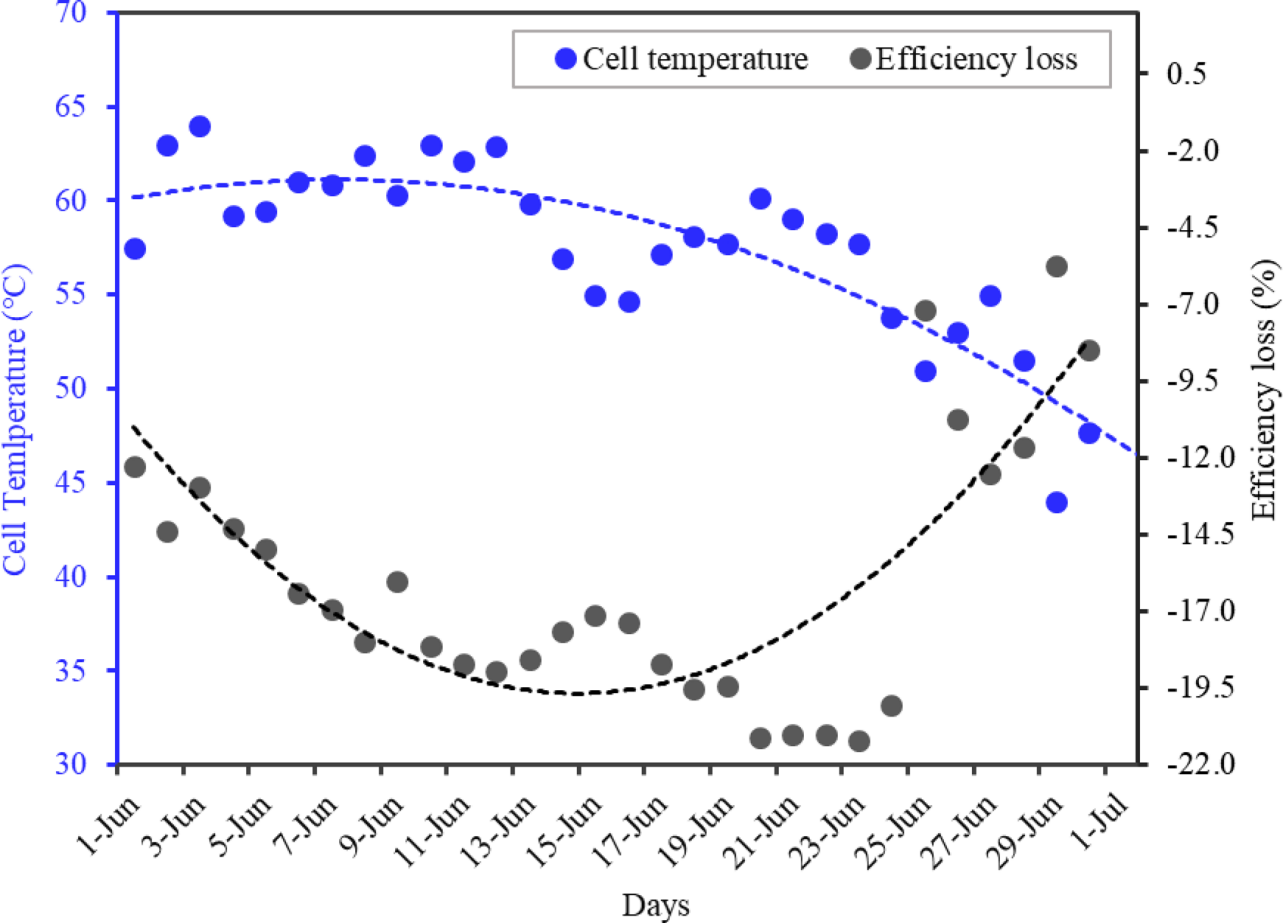
Variation in PV system efficiency due to increment of solar PV module temperature.

As shown in the Fig. 12, the maximum loss in efficiency was recorded as 20.4% on June 23 compare to the other days of the month. The maximum cell temperature was recorded 64 °C for the month. The monthly averaged daily loss in efficiency of the module was found 16.0%. The minimum reduction in the efficiency was found to be 4.8%, recorded on 29th of June. This occurred due to abundant rain on the day that caused decline in ambient temperature and thus resulting in cell temperature decrease. The minimum loss in the efficiency was recorded for the last 5 days of the month for which the average efficiency loss was below 10.0%. Figure 12 also shows the efficiency loss characteristics of the SPV system and it suggests that the temperature-rise directly reduces the on-field efficiency of SPV system. Further, it is also indicates that other factors, such as dust buildup, are responsible for the efficiency decline even at lower temperatures.

### Convective heat transfer and PV power loss

**Fig. 13:**
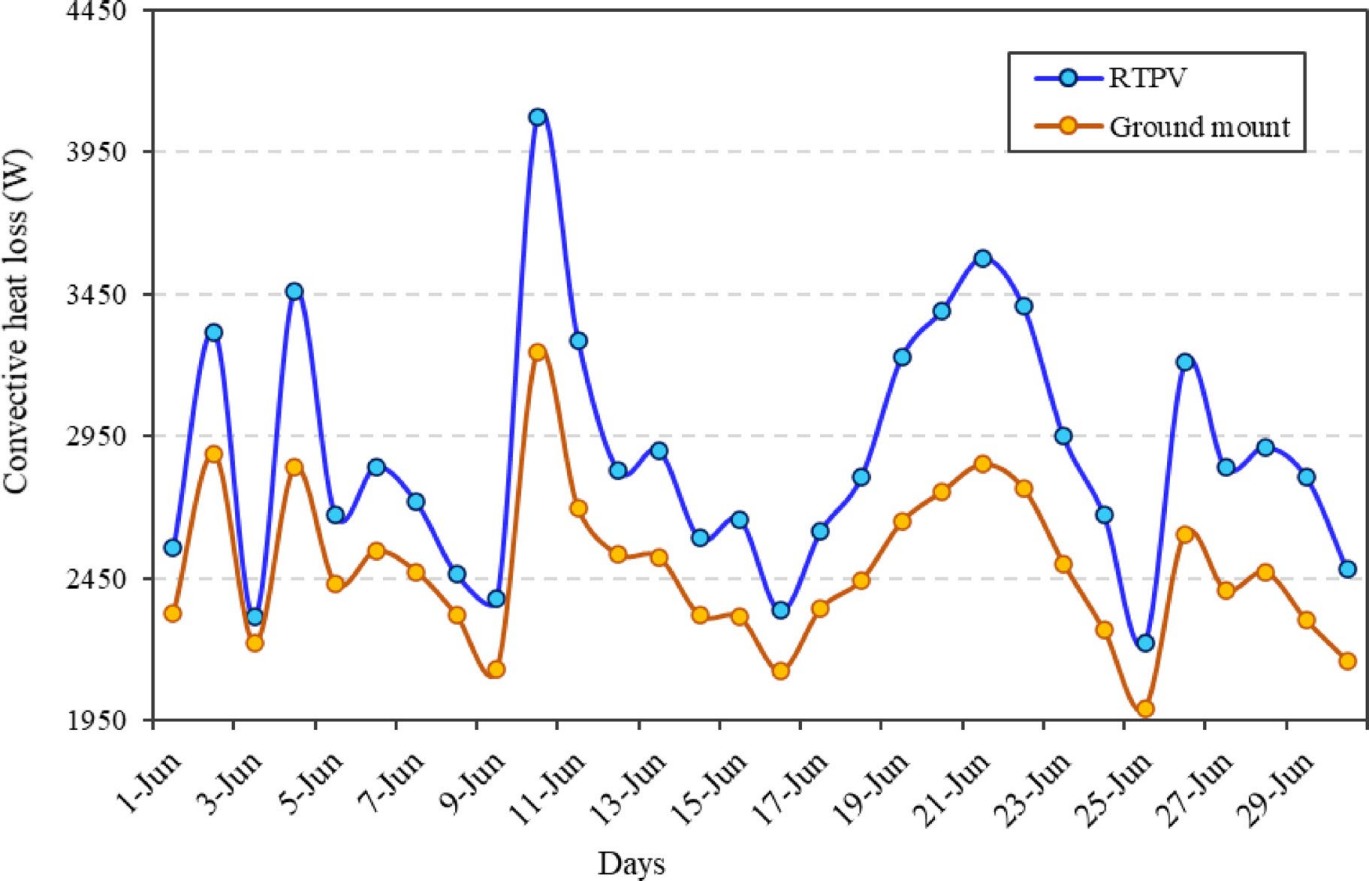
Daily convective heat loss comparison between RTPV and ground-mounted PV systems during June under composite climate conditions.

Good wind speed provides convective cooling of solar PV modules, which is a critical factor in maintaining their efficiency, as it helps to regulate their operating temperature^37^. The study examines the wind-driven convective cooling effect on the performance of the RTPV system. For this, Eq. 12 was used to calculate the convective heat loss for the RTPV system and its results were compared to those of a simulated ground-mounted PV system, with the same climatic condition, as shown in Fig. 13.

Figure shows that a higher wind speed (> 8 m/s) lowers the cell temperature by approximately 4 °C thereby indicating an inverse relationship between the wind speed and cell temperature. It is evident that the RTPV system consistently experiences higher convective heat loss compared to the ground-mounted system, indicating more effective cooling on the rooftop. This trend is primarily attributed to higher wind velocities at elevated rooftop levels, which enhance air movement and thus increase heat dissipation. The convective heat loss for RTPV varies approximately between 2,400 W and 4,050 W, while for the ground-mounted system, it ranges from about 2,200 W to 3,200 W. A notable peak occurs on June 11, where the RTPV system reaches its highest loss of around 4,050 W, approximately 900 W more than the ground-mounted system on the same day. Dips in convective loss are observed around June 17 and June 26, likely due to rain or reduced temperature gradients. The RTPV system exhibited a 16.19% higher convective heat loss, indicating more effective cooling due to higher average wind velocity, which was 8.9 m/s compared to 4.7 m/s for ground mount. RTPV records a gain of 56.14% in the wind velocity. This enhanced airflow over the rooftop modules helped maintain a lower average cell temperature as 57.6 °C compared to 62.9 °C for ground mount, representing a 8.59% reduction compared to the ground-mounted setup. The comparison further reveals the compensatory role of convective cooling: average rooftop wind speed of 8.9 m/s reduced module temperatures compared to a simulated ground-mounted system with 4.7 m/s wind speed. This enhanced convective heat loss partially offset radiative heating, highlighting the importance of site-specific wind exposure in mitigating thermal effects. Overall, the data highlight the thermal advantage of RTPV systems, where stronger convective cooling contributes to lower module temperatures and improved system efficiency.

**Fig. 14:**
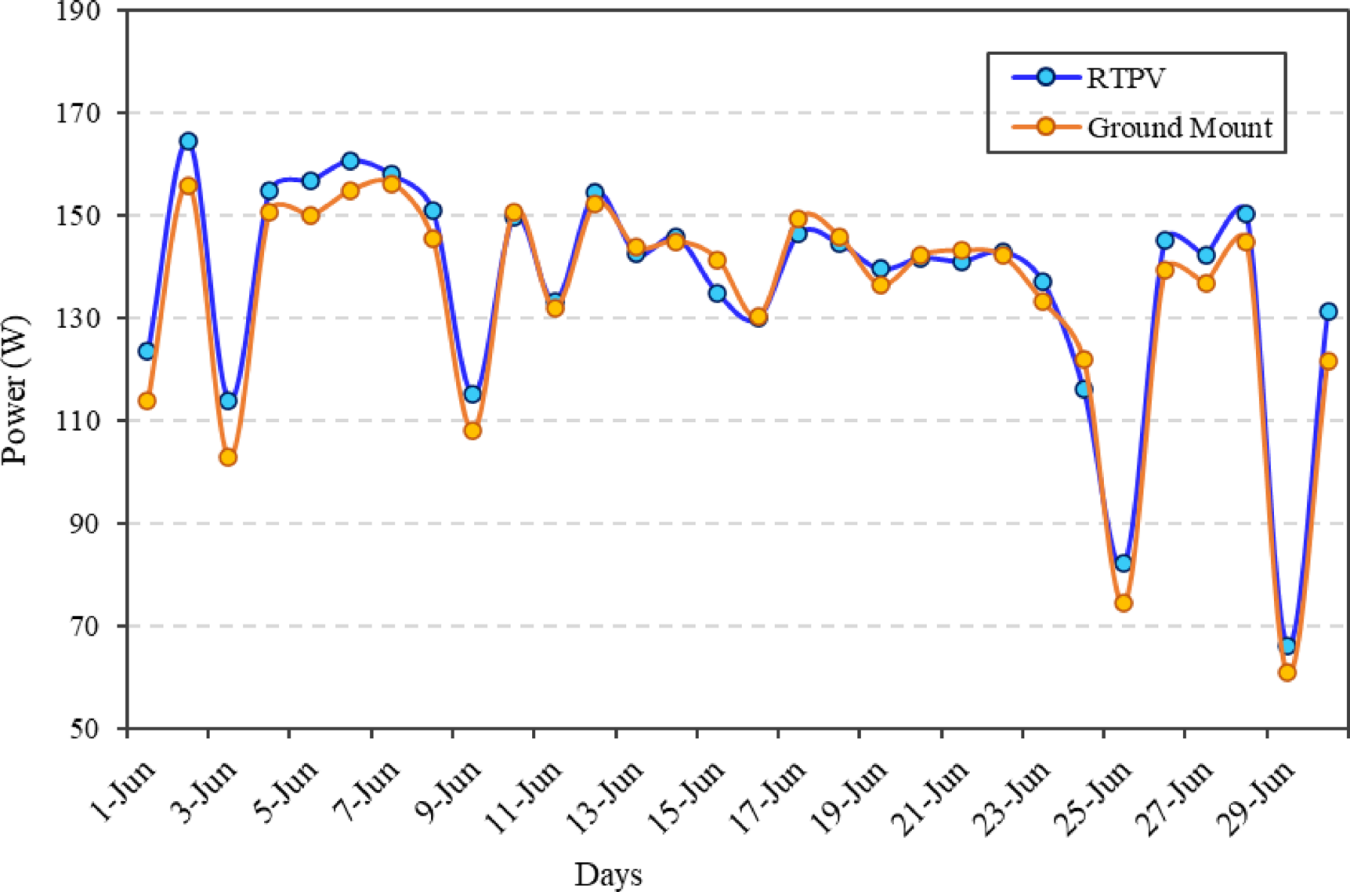
Daily power output loss comparison between RTPV and ground-mounted PV systems during June under composite climate conditions.

Figure 14 displays the daily power output of RTPV and ground-mounted PV systems over the month of June. Both systems exhibit a similar overall trend in power generation, reflecting the influence of common weather conditions like solar irradiance, temperature and rainfall. However, RTPV consistently records marginally higher output than the ground-mounted system on most days, indicating the beneficial effect of enhanced convective cooling on the rooftop.

Power output ranges from about 65 W to 170 W for both configurations, with peak generation occurring on 3rd June and 6th June, where RTPV slightly outperforms ground-mounted modules. Significant dips are observed on 9th June, 25th June, and 29th June, due to overcast or rainy days, which reduce exposure to solar irradiance and PV module yield. Despite these fluctuations, enhanced cooling through increased wind exposure enabled the RTPV system to demonstrate a consistent increase in power output compared to the ground-mounted system. Specifically, the system generated 4,113 W cumulative power over the month compared to 4,022 W from the ground-mounted system, reflecting a 2.27% higher yield. Additionally, the temperature-corrected average efficiency of the RTPV system was 10.90%, slightly higher than the ground-mounted system’s 10.70%, reflecting a 1.87% improvement. Results illustrates that improved convective cooling on RTPV modules leads to marginally better thermal regulation and power generation. The slight advantage in RTPV performance aligns with earlier findings of higher wind speeds and lower cell temperatures, which help to reduce thermal losses^28^.

### Effect of soiling on PV performance

**Fig. 15:**
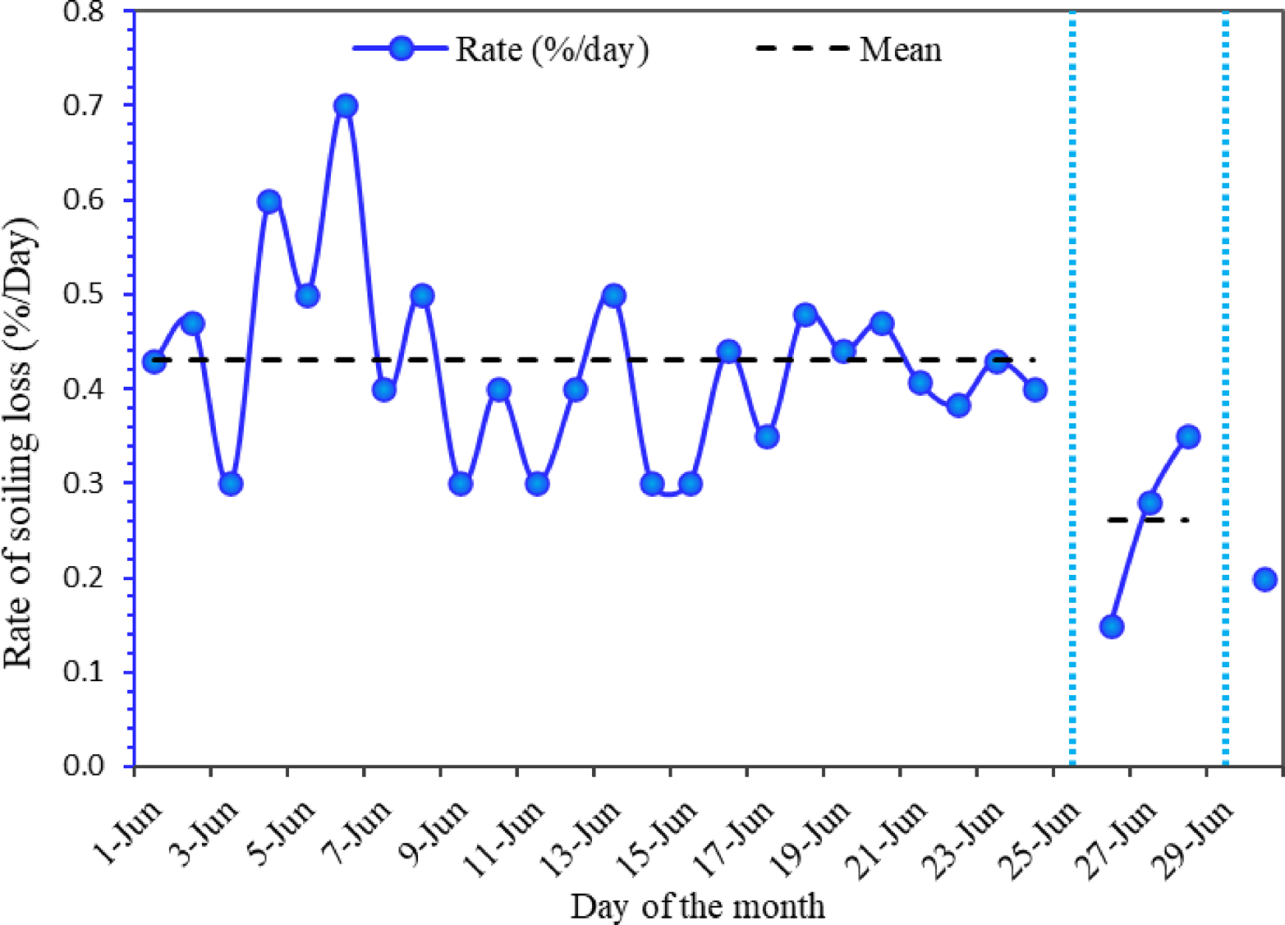
Daily soiling loss rate (%), calculated as the difference in soiling loss between consecutive days for the month.

We cannot prevent the module from becoming dirty because it operates in an open environment. The soiling of the present PV system was analysed by using Eq. 14. Daily system efficiency (η_real_) was calculated with the help of daily produced power and in plane solar radiation. Figure 15 displays the rate of soiling loss, which has been calculated by subtracting one day soiling loss to the next day for the whole month. Soiling loss ranges from 0.20% to 0.70% per day. Higher rate was recorded during dry days such as from 1 st June to 25th June. This time spell shows that deposition of dust increases without rainfall. Before rainfall average soiling loss was recorded about 0.43% per day and after it was 0.26% per day. Raining events on 25th June (10.92 mm) and June 29th (55.12 mm) completely cleaned the panel surface. The results shows the importance of natural rainfall in restoring PV modules yield in high soiling regions.

**Fig. 16:**
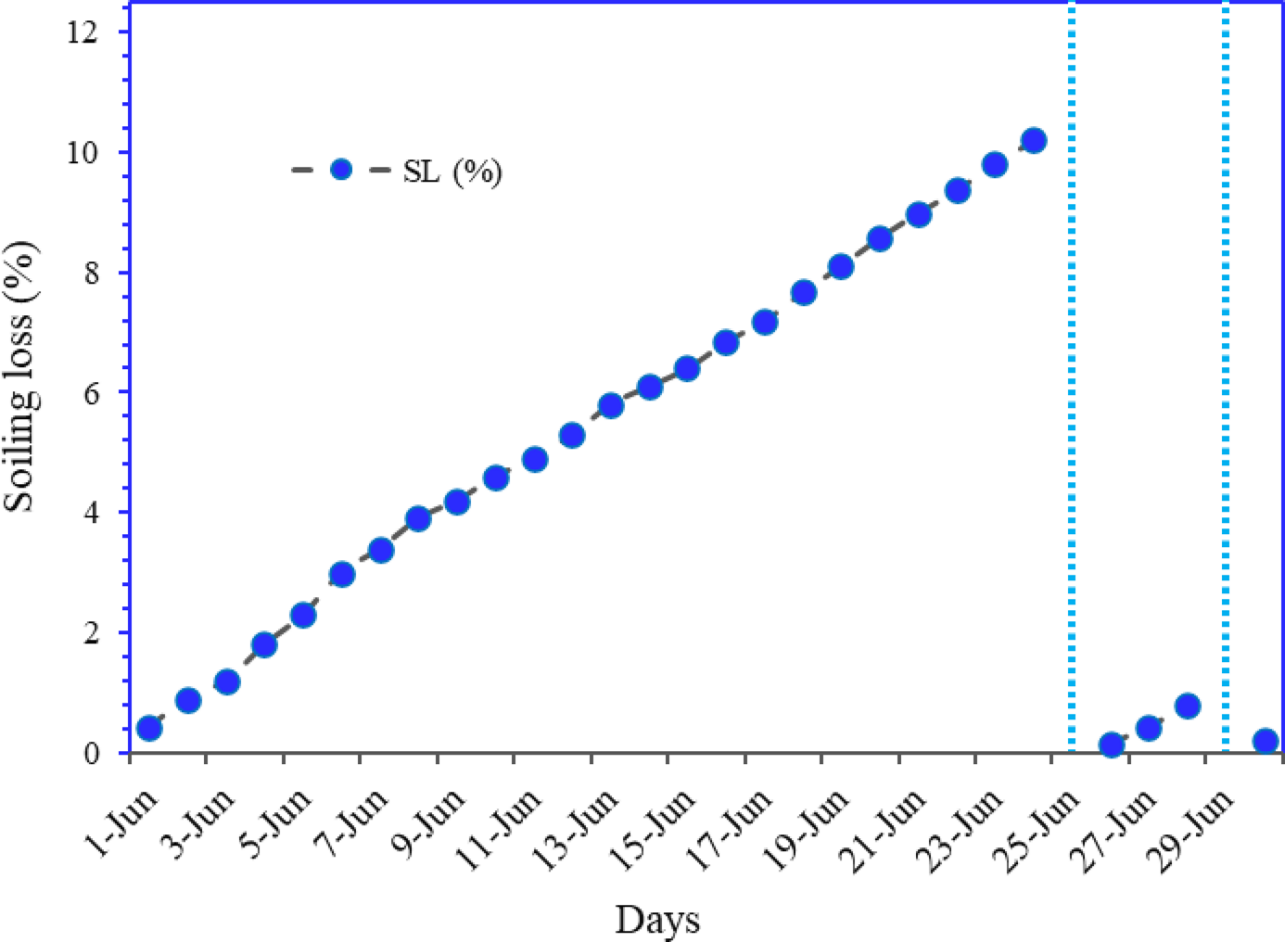
Variation of soiling loss and rain (mm) over the month of June.

Figure 16 illustrates the variation in soiling loss and rainfall (mm) over the month. Soiling loss increases steadily from around 0.43% on June 1 st and the highest cumulative soiling loss was recorded at 10.2% on the 24th of June indicating significant dust accumulation on solar PV modules. Minimal rainfall before June 25 fails to clean the panels, leading to rising soiling loss. However, heavy rainfall events on June 26 (> 10 mm) and June 29 (> 50 mm) sharply reduced the soiling loss to nearly 0%, demonstrating the natural cleaning effect of rain. Soiling is a complex procedure to analyze due to the cumulative effect of weather parameters^8^. Wind increases dust precipitation on the module surface, and high wind also acts as a dust remover for the modules^38^. Heavy rainfall works as a natural cleaner of solar PV modules and suspended particulates from the atmosphere^39^. The figure highlights how soiling loss degrades PV performance over time and how rainfall effectively restores efficiency.

**Fig. 17:**
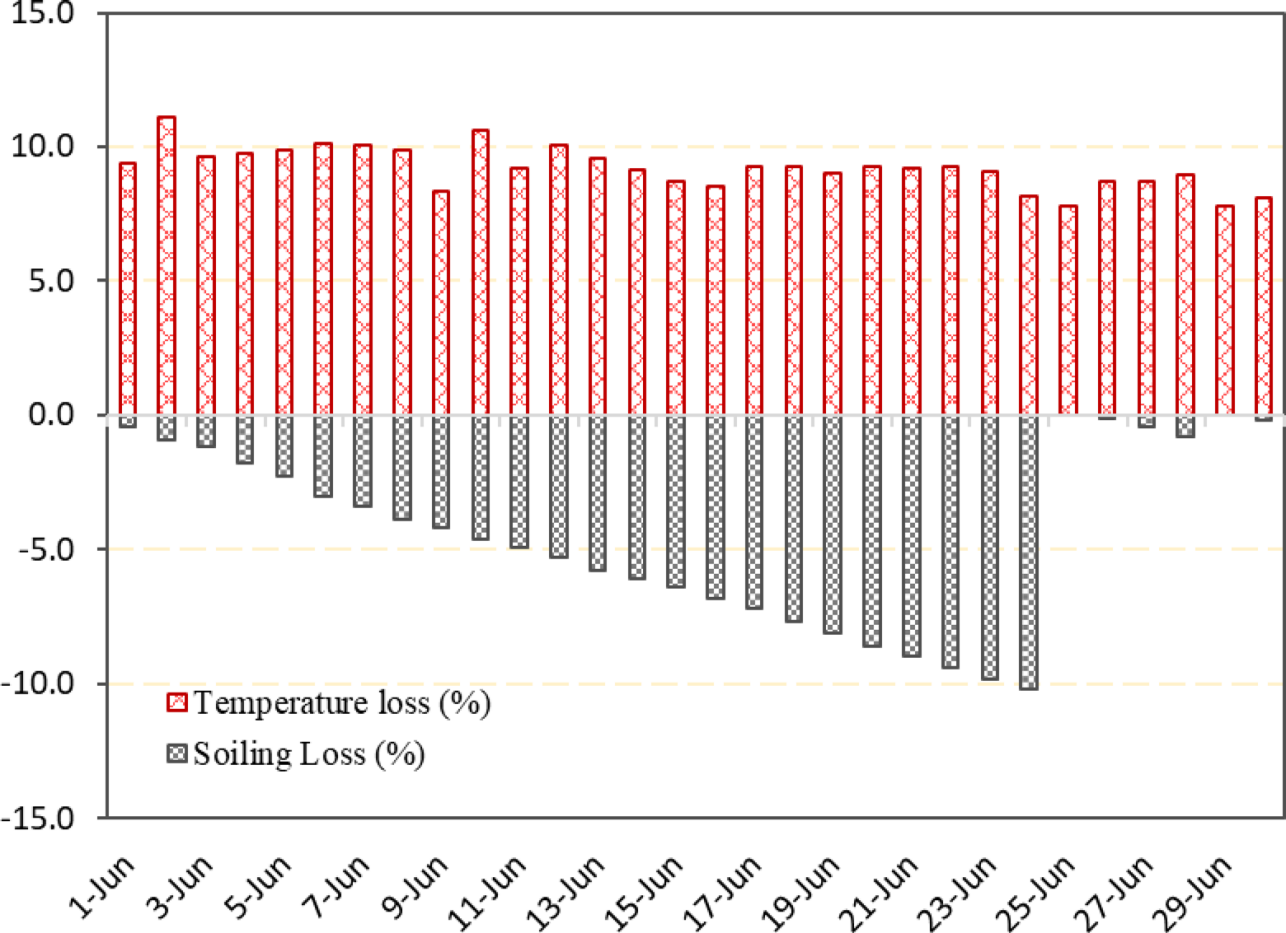
Percentage power loss due to temperature and soiling over June, showing combined and the restorative effect of rainfall.

Figure 17 shows the total power loss due to elevated temperature and soiling condition over the month of June. Due to higher module temperatures, the system experienced an output power drop ranging from 7.6% to 15.2%. However soiling on PV modules reduces output power ranging from 0% to 10.2%. The same trend was recorded in dry and semi-arid zone of North India showing monthly soiling losses up to 11.7% per month in a rooftop PV system, with higher losses during dry, dust-intensive seasons and lower losses during monsoons due to natural rain cleaning^40^. A similar reduction was observed in monthly energy output of 5.6% for 7.5% soiling and 10.8% for 12.5% soiling for the SPV installation in the deserts of Oman. The authors reported that energy yield degradation reaching 18.1% after three weeks and 38.1% after five weeks, confirming the compounding effects of prolonged dust accumulation^41^. Matsumoto et al. (2020), who observed a 12–14% monthly power degradation due to natural soiling on a 60 kWp system in Mexico City^42^. Micheli et al. (2022) observed soiling losses between 5% and 11% in a utility-scale PV system in Chile, with variations due to nonuniform soiling patterns^43^ . A study reviewed global soiling trends and reported soiling losses ranging from 6.5% in two months under moderate conditions to up to 70% in extreme desert environments^9^.

Figure suggests that rain recovered PV performance about 100% as seen June 25 and 29. Similar results observed in the lots of previous studies, where rain reduced soiling loss upto 0%^8,44^. PV module temperature recorded high throughout the month resultant an average loss of 7.5% which is typical for composite climate regions. Similarly a reduction in power by 7.01% and 6.68% was claimed for series and parallel connected m-si solar cells^45^. Rain also puts positive impact on the module’s operating temperature by decreasing it^46^. As figure suggest after rainfall reduction in temperature loss was recorded due to reduced ambient temperature.

These reductions due to elevated temperature and soiling highlight the need for scheduled cleaning to recover PV performance. A study demonstrates that regular cleaning and water-based cooling significantly enhance the performance of photovoltaic panels in high-temperature, dusty environments, with energy output improving by up to 23.9%^47^. The study highlights the role of passive wind cooling in reducing PV module temperature, which aligns with findings from Goossens et al.^35^. However, active cooling techniques, such as water-based systems (sprays, circulating fluids), forced air cooling (fans), or phase-change materials (PCMs), offer controlled and potentially greater temperature reduction. These active systems circulate coolant media—typically air, water, or nanofluids—to absorb and remove excess heat from PV panels. Water-based active cooling methods, including spray nozzles and thin-film water flow, have shown impressive temperature drops of up to 25 °C, resulting in electrical efficiency gains of 14–17% under intense solar radiation^48^. For instance, water cooling can lower cell temperatures by 10–20 °C, improving efficiency by 5–12%^5^, but requires energy for pumps and water management. PCMs absorb latent heat without moving parts, offering ~ 8% efficiency gains^49^, but their effectiveness depends on material properties and ambient conditions.

In contrast, passive wind cooling is energy-free and maintenance-light but relies on variable environmental factors. Active methods provide consistent cooling but increase system complexity, cost, and energy consumption. For the studied site, passive cooling suffices due to high wind speeds, but hybrid approaches (e.g., combining wind-driven convection with intermittent water sprays) could optimize performance in low-wind or high-soiling regions. Future work will explore these synergies. Applying various active and passive cooling techniques, along with both automatic and manual cleaning methods, can improve the on-field performance of solar PV systems^50^.

### Comparative analysis and key findings

The preceding analysis detailed the individual effects of temperature, soiling, and wind on the rooftop PV system’s performance. To synthesize these findings, Table 4 provides a consolidated summary of the key performance metrics, directly comparing the rooftop installation with a simulated ground-mounted system under the same climatic conditions.

**Table 4:** Summary of key performance metrics for rooftop and simulated ground-mounted PV systems.

S. No.	Performance Metric	Rooftop PV (RTPV)	Ground-Mounted PV (Simulated)	Relative Change & Implication
1.	Average Cell Temperature (T_C_)	57.5 °C	62.9 °C	8.59% for RTPV, indicating better cooling.
2.	Peak Cell Temperature	64.0 °C	76.88 °C	8.59% for RTPV, Highlights thermal stress mitigation on rooftop
3.	Average Wind Speed	8.9 m/s	4.7 m/s	+ 89.4% for RTPV, confirming enhanced exposure.
4.	Average Convective Heat Loss	2885 W	2483 W	+ 16.2% for RTPV, quantifying the cooling benefit
5.	Average PV module efficiency	10.9%	10.7%	1.8% gain for RTPV, direct result of better cooling
6.	Average power generation	137 W	134 W	+ 2.2% for RTPV, net energy gain.
7.	Average Soiling Loss Rate (Dry Period)	0.43%/day	Assumed similar	N/A, emphasizes need for cleaning schedule.
8.	Cumulative Soiling Loss	10.2%	Assumed similar	N/A, Shows significant soiling loss during dry days.

## Conclusion

This study experimentally analyzed the combined impact of elevated operating temperatures and soiling on the performance of a 500 Wp grid-connected rooftop PV system in Lucknow, India - a region characterized by composite climatic conditions. The results demonstrated that cell temperatures reaching up to 64.0 °C during peak solar hours led to an efficiency loss of 12.0%, with maximum losses of 20.4% observed under combined thermal stress and soiling. Simultaneously, soiling accumulation rates averaged 0.43%/day, culminating in a 10.2% monthly power loss in the absence of rainfall. Notably, natural rainfall events reduced soiling losses to 0.26%/day and lowered cell temperatures by up to 15 °C, highlighting their dual role as both a cleaning agent and a passive cooling mechanism. Furthermore, the study highlights the thermal advantage of rooftop PV systems over ground-mounted installations due to higher convective heat loss, facilitated by stronger wind exposure. The rooftop configuration exhibited a 2.27% higher energy yield and a 6.45% reduction in average cell temperature compared to its ground-mounted counterpart.

Based on our findings, we recommend that solar PV installations in regions with climatic conditions similar to North India should integrate regular maintenance schedules, especially during dry months, to mitigate soiling losses. Incorporating simple cleaning mechanisms or low-cost automated systems could significantly improve energy output. Additionally, selecting module tilt angles optimized not just for irradiance but also for minimizing dust accumulation can further enhance performance. Future research should evaluate the effectiveness of various anti-soiling coatings under real-world conditions and explore machine learning-based models to forecast performance loss due to environmental factors. Long-term studies analysing the combined impact of temperature, humidity, and soiling on PV performance would also provide valuable insights for improving system design and energy forecasting accuracy.

## Data Availability

The datasets during and/or analyzed during the current study are available from the corresponding author upon reasonable request.

## Abbreviations

GI: Galvanized iron
GHI: Global Horizontal Irradiance
G_T_: Plane of array irradiance (W/m²)
H_d_: Daily diffuse insolation (kWh/m²/day)
H: Global horizontal insolation (kWh/m²/day)
H_O_: Extraterrestrial radiation on horizontal surface (kWh/m²/day)
H_T_: Total insolation on PV module (kWh/m²/day)
I_SC_: Short-circuit current (A)
K_T_: Sky clearness index
MERRA: Modern-Era Retrospective analysis for Research and Applications
NREL: National Renewable Energy Laboratory
P_mp_: Power at maximum power point (W)
P_real_: Recorded power (W)
P_th_: Theoretical PV power (W)
PVUSA: Photovoltaics for Utility Scale Applications
RTPV: Rooftop Photovoltaic
R_b_: Geometric factor for beam radiation
SL: Soiling loss (%)
SPV: Solar Photovoltaic
STC: Standard Test Conditions
T_a_: Ambient temperature (°C)
T_C_: Cell temperature (°C)
U_C_: Constant heat loss factor (W/m²K)
U_V_: Wind loss factor (W/m²K·m/s)
V_OC_: Open-circuit voltage (V)
Wv: Wind velocity (m/s)

## Greek symbols

α: Temperature coefficient of power (%/°C)
β: Tilt angle of PV module (°)
δ: Solar declination angle (°)
ρ_g_: Ground reflectance
φ: Latitude of the site (°)
ω: Hour angle (°)
